# Supplementary material for: Insulated transcriptional elements enable precise design of genetic circuits
Source: Nat Commun. 2017 Jul 3;8:52. doi: 10.1038/s41467-017-00063-z (PMC5495784; doi:10.1038/s41467-017-00063-z)
Supplement: Supplementary file 1 — Supplementary Information [file 41467_2017_63_MOESM1_ESM.pdf]

File Name: Supplementary Information
Description: Supplementary Figures, Supplementary Tables and Supplementary References

File Name: Peer Review File
Description:

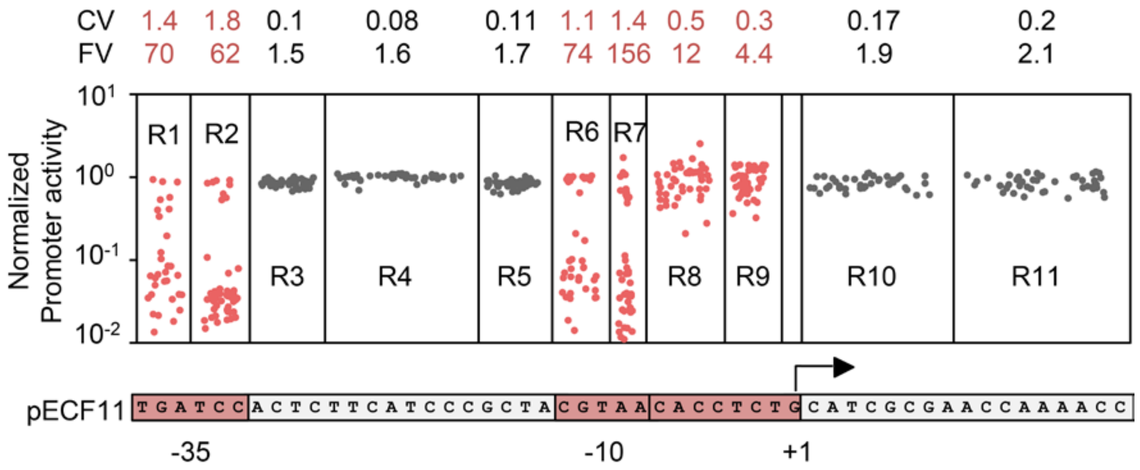

Supplementary Figure 1. Segment-by-segment saturation mutagenesis of P_{ECF11} on a medium-copy-number backbone.

Each dot represents a randomly selected mutant containing mutations in the corresponding sequence segment. The promoter activity was measured using superfolder GFP as the reporter and quantified as the arithmetic mean of flow cytometry fluorescence data. CV, coefficient of variation; FV, relative (-fold) variation determined by the ratio of maximal promoter activity to the minimal observed value. Data represent the averages of at least three replicate experiments conducted on different days.

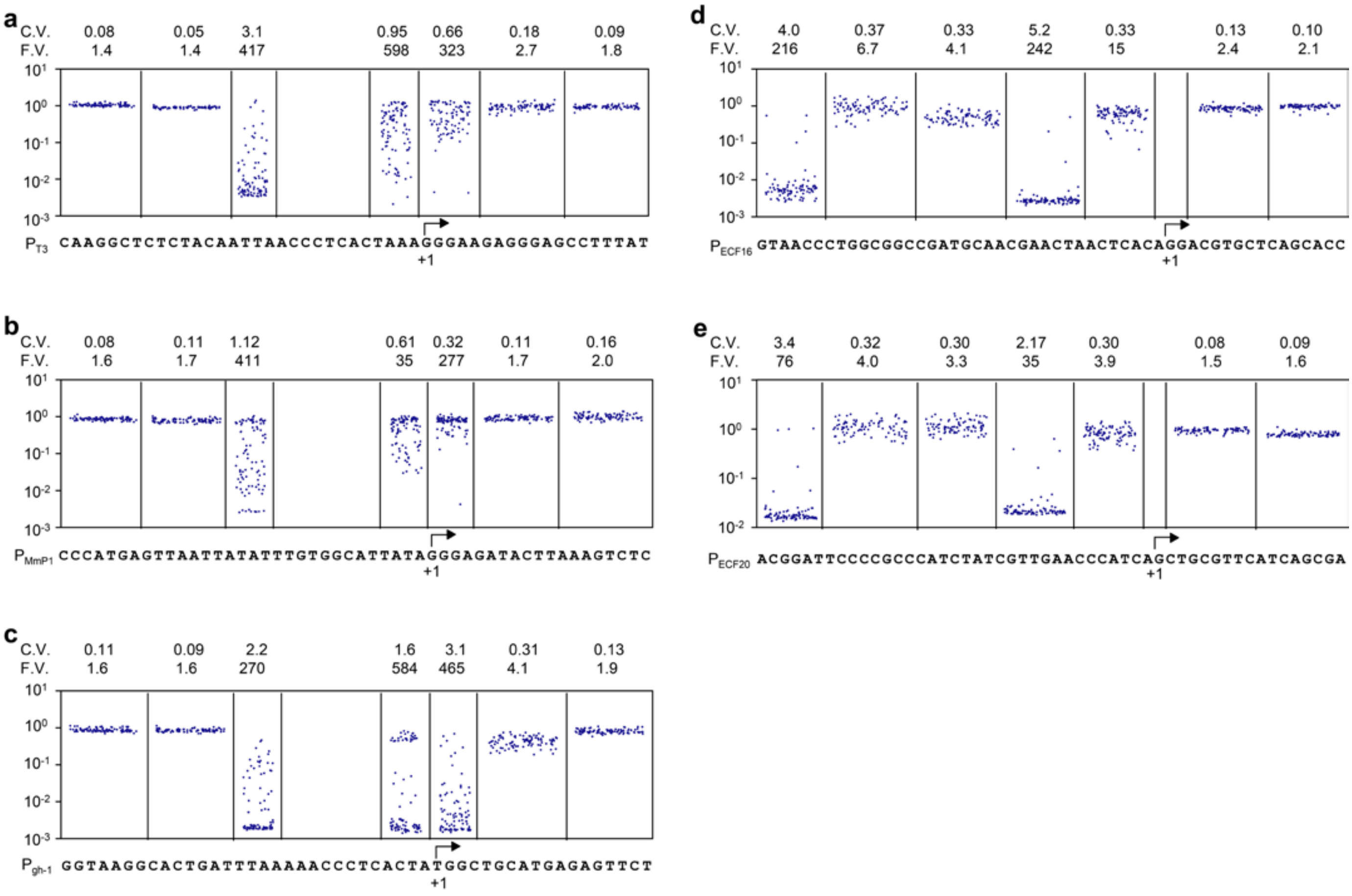

Supplementary Figure 2. Genetic Refinement of Additional ECF and T7-Family Promoters. (**a**) Effect of random mutagenesis on the promoter activity of P_{T3}. (**b**) Effect of random mutagenesis on the promoter activity of P_{MmP1}. (**c**) Effect of random mutagenesis on the promoter activity of $P_{gh\text{-}1}$. (**d**) Effect of random mutagenesis on the promoter activity of P_{ECF16}. (**e**) Effect of random mutagenesis on the promoter activity of P_{ECF20}. Each dot represents a randomly selected mutant containing mutations in the corresponding sequence segment. The promoter activity was measured using superfolder GFP as the reporter and quantified as the arithmetic mean of flow cytometry fluorescence data. CV, coefficient of variation; FV, relative (-fold) variation determined by the ratio of maximal promoter activity to the minimal observed value. Data represent the averages of at least three replicate experiments conducted on different days.

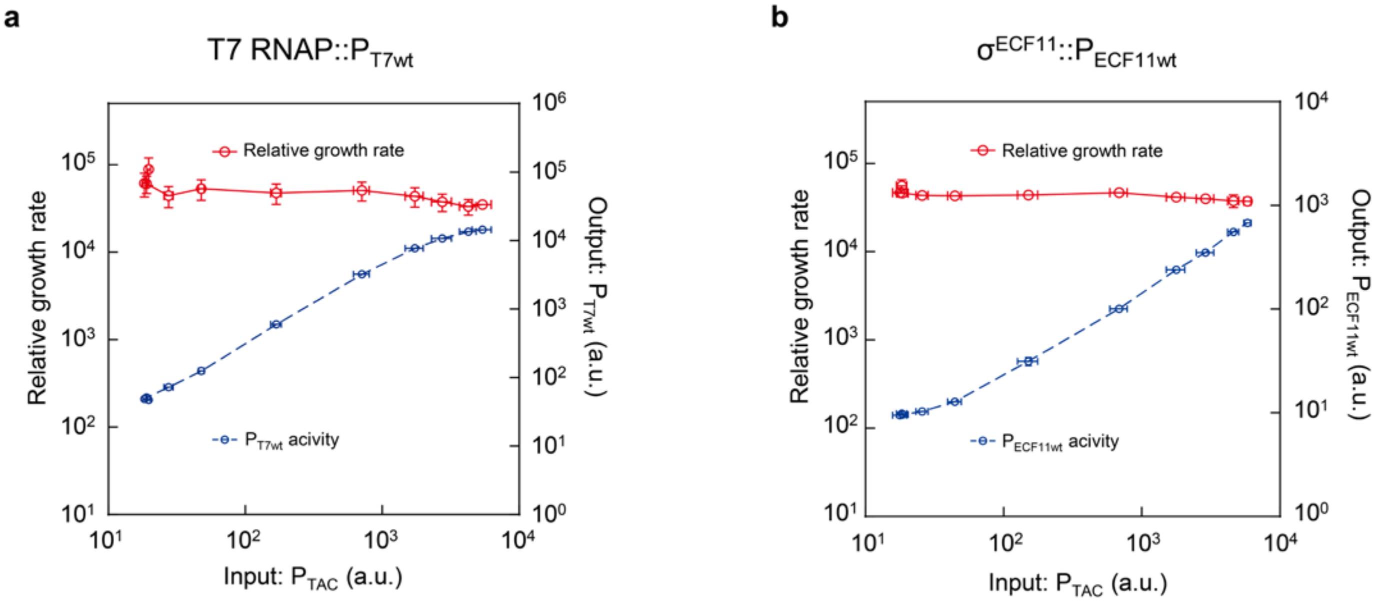

Supplementary Figure 3. Effects of activator expressions on the growth rate of *E.coli*. (**a**) Effect of T7 RNAP and its sfGFP reporter. (**b**) Effect of σ^{ECF11} and sfGFP reporter. The relative growth rate was obtained by extracting the “cell event number per ten seconds” from the flow cytometry data of each measurement. Data represent the means ± SD from at least three replicate experiments conducted on different days.

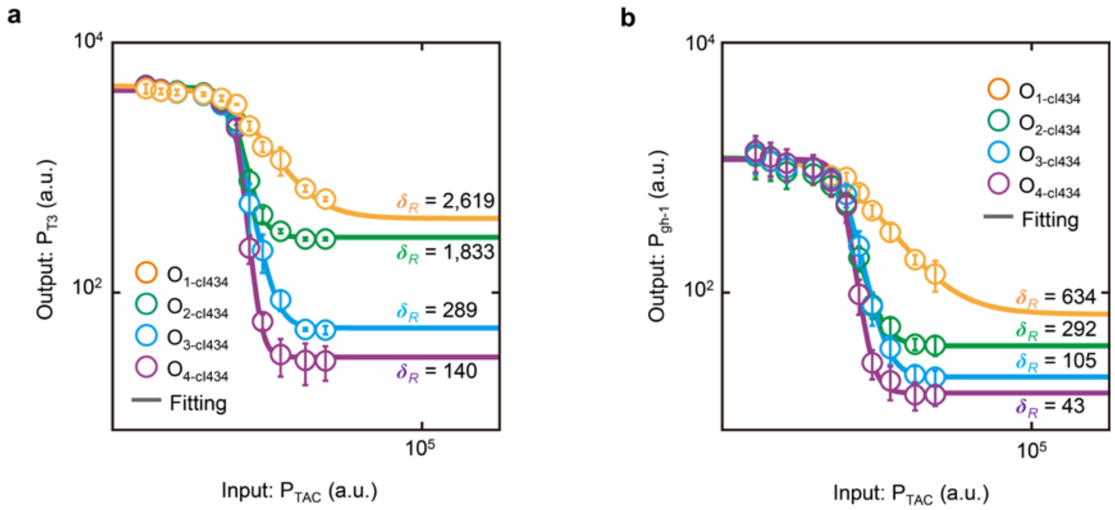

Supplementary Figure 4. Experimental Measurements and Parameter Fitting of Response Functions for cI434 acting on P_{T3} (a) and $P_{gh\text{-}1}$ (b). Solid lines represent the data of parameter fitting using a non-equilibrium correction term. Open circuits represent experimental measurements. δ_R values for each response function obtained from model fitting are given. The promoter activity was calculated as the arithmetic mean of flow cytometry fluorescence data using sfGFP as the reporter. Data represent the means ± SD from at least three replicate experiments conducted on different days.

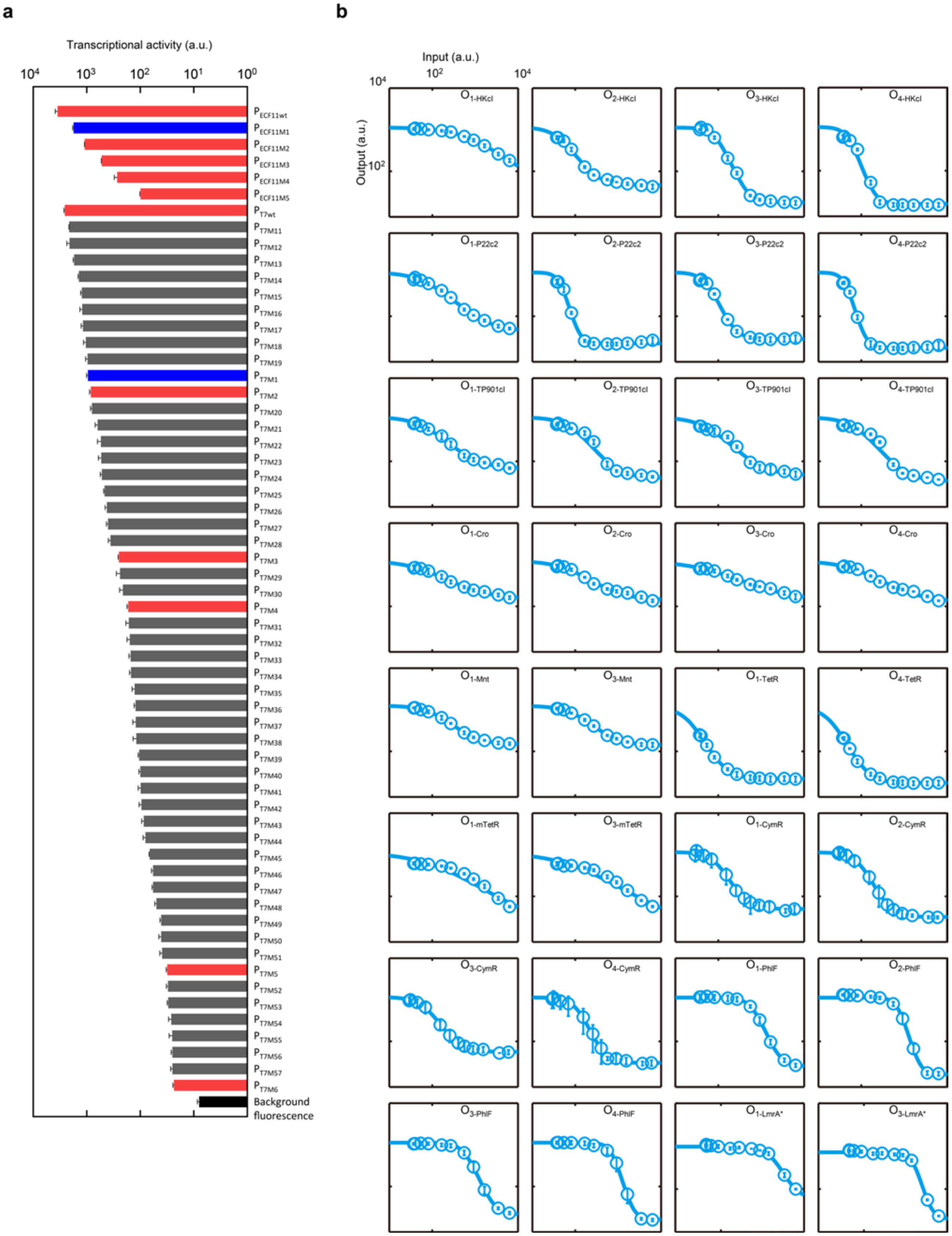

Supplementary Figure 5. Raw Data and Model Fitting for T7-RNAP Promoter Cores and 32 Repressor-Operator Pairs. (**a**) Transcriptional activity of T7-RNAP and ECF11 promoter cores. Promoter cores described in Fig. 4 are highlighted in red and those used for the fitting of repression-dependent parameters are highlighted in blue. The corresponding sequences of the promoter cores are listed in Supplementary Table 3. (**b**) Experimental measurements and parameter fitting of response functions for 32 repressor-operator pairs. The response functions for the four operators targeted by cI434 are shown in Fig. 3. Open circles and solid lines represent experimental data and model fitting results, respectively. Data represent the means ± SD from at least three replicate experiments conducted on different days.

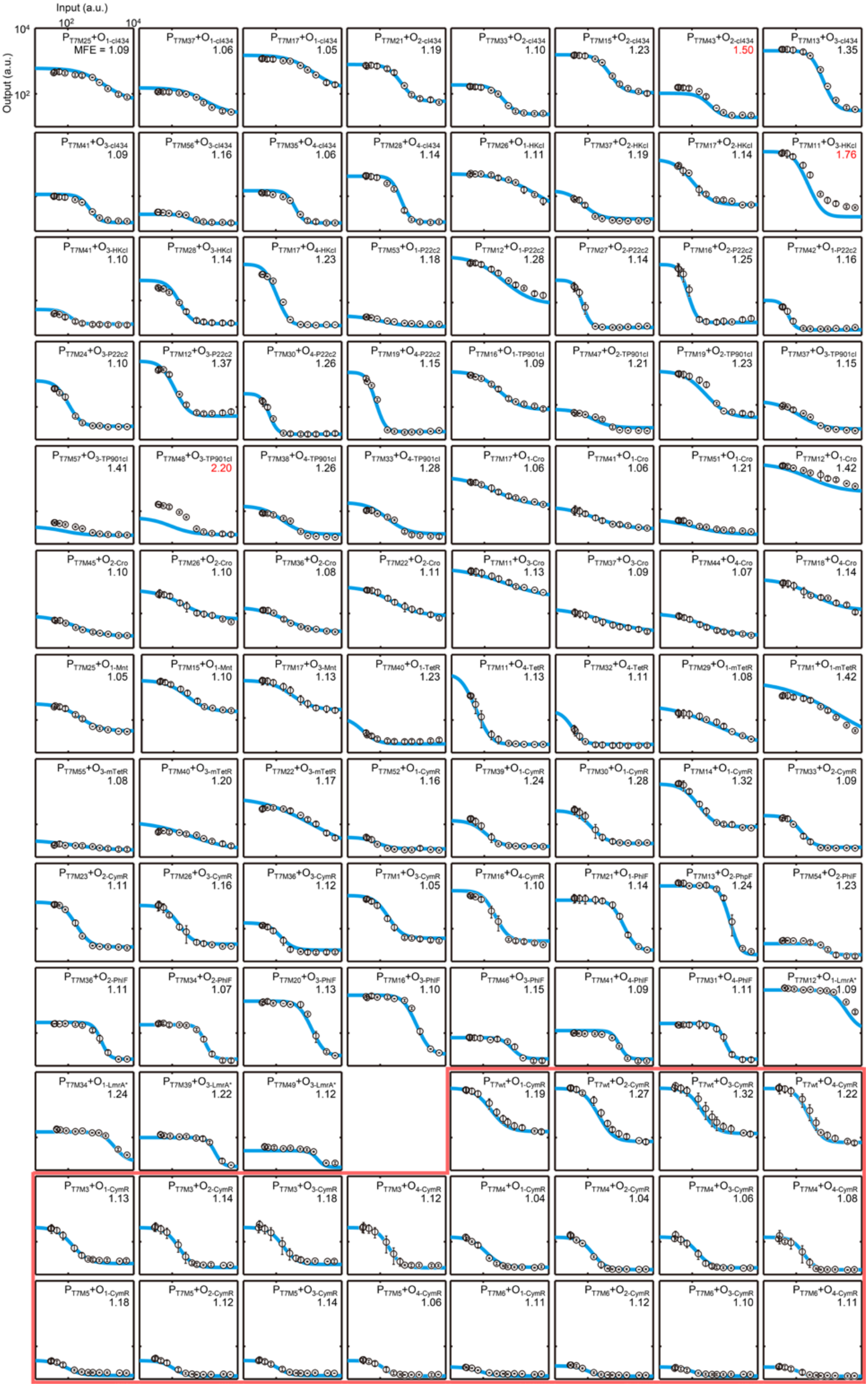
Input (a.u.)
10^2
10^4
Output (a.u.)
10^4
10^2
PT7M25+O1-cI434 MFE = 1.09
PT7M37+O1-cI434 1.06
PT7M17+O1-cI434 1.05
PT7M21+O2-cI434 1.19
PT7M33+O2-cI434 1.10
PT7M15+O2-cI434 1.23
PT7M43+O2-cI434 1.50
PT7M13+O3-cI434 1.35
PT7M41+O3-cI434 1.09
PT7M56+O3-cI434 1.16
PT7M35+O4-cI434 1.06
PT7M28+O4-cI434 1.14
PT7M26+O1-HKcI 1.11
PT7M37+O2-HKcI 1.19
PT7M17+O2-HKcI 1.14
PT7M11+O3-HKcI 1.76
PT7M41+O3-HKcI 1.10
PT7M28+O3-HKcI 1.14
PT7M17+O4-HKcI 1.23
PT7M53+O1-P22c2 1.18
PT7M12+O1-P22c2 1.28
PT7M27+O2-P22c2 1.14
PT7M16+O2-P22c2 1.25
PT7M42+O1-P22c2 1.16
PT7M24+O3-P22c2 1.10
PT7M12+O3-P22c2 1.37
PT7M30+O4-P22c2 1.26
PT7M19+O4-P22c2 1.15
PT7M16+O1-TP901cI 1.09
PT7M47+O2-TP901cI 1.21
PT7M19+O2-TP901cI 1.23
PT7M37+O3-TP901cI 1.15
PT7M57+O3-TP901cI 1.41
PT7M48+O3-TP901cI 2.20
PT7M38+O4-TP901cI 1.26
PT7M33+O4-TP901cI 1.28
PT7M17+O1-Cro 1.06
PT7M41+O1-Cro 1.06
PT7M51+O1-Cro 1.21
PT7M12+O1-Cro 1.42
PT7M45+O2-Cro 1.10
PT7M26+O2-Cro 1.10
PT7M36+O2-Cro 1.08
PT7M22+O2-Cro 1.11
PT7M11+O3-Cro 1.13
PT7M37+O3-Cro 1.09
PT7M44+O4-Cro 1.07
PT7M18+O4-Cro 1.14
PT7M25+O1-Mnt 1.05
PT7M15+O1-Mnt 1.10
PT7M17+O3-Mnt 1.13
PT7M40+O1-TetR 1.23
PT7M11+O4-TetR 1.13
PT7M32+O4-TetR 1.11
PT7M29+O1-mTetR 1.08
PT7M1+O1-mTetR 1.42
PT7M55+O3-mTetR 1.08
PT7M40+O3-mTetR 1.20
PT7M22+O3-mTetR 1.17
PT7M52+O1-CymR 1.16
PT7M39+O1-CymR 1.24
PT7M30+O1-CymR 1.28
PT7M14+O1-CymR 1.32
PT7M33+O2-CymR 1.09
PT7M23+O2-CymR 1.11
PT7M26+O3-CymR 1.16
PT7M36+O3-CymR 1.12
PT7M1+O3-CymR 1.05
PT7M16+O4-CymR 1.10
PT7M21+O1-PhlF 1.14
PT7M13+O2-PhpF 1.24
PT7M54+O2-PhlF 1.23
PT7M36+O2-PhlF 1.11
PT7M34+O2-PhlF 1.07
PT7M20+O3-PhlF 1.13
PT7M16+O3-PhlF 1.10
PT7M46+O3-PhlF 1.15
PT7M41+O4-PhlF 1.09
PT7M31+O4-PhlF 1.11
PT7M12+O1-LmrA* 1.09
PT7M34+O1-LmrA* 1.24
PT7M39+O3-LmrA* 1.22
PT7M49+O3-LmrA* 1.12
PT7wt+O1-CymR 1.19
PT7wt+O2-CymR 1.27
PT7wt+O3-CymR 1.32
PT7wt+O4-CymR 1.22
PT7M3+O1-CymR 1.13
PT7M3+O2-CymR 1.14
PT7M3+O3-CymR 1.18
PT7M3+O4-CymR 1.12
PT7M4+O1-CymR 1.04
PT7M4+O2-CymR 1.04
PT7M4+O3-CymR 1.06
PT7M4+O4-CymR 1.08
PT7M5+O1-CymR 1.18
PT7M5+O2-CymR 1.12
PT7M5+O3-CymR 1.14
PT7M5+O4-CymR 1.06
PT7M6+O1-CymR 1.11
PT7M6+O2-CymR 1.12
PT7M6+O3-CymR 1.10
PT7M6+O4-CymR 1.11

Supplementary Figure 6. Experimentally measured and predicted response functions of 107 Combinational Promoters Selected for Genetic Implementation. Boxed in red are the combinational promoter designs using cymR as the repressor which were selected as a group. Others were selected randomly. Mean relative (-fold) errors for each response function and the corresponding promoter design are given. The promoter activity was measured using superfolder GFP as the reporter and quantified as the arithmetic mean of flow cytometry fluorescence data. Data represent the means ± SD from at least three replicate experiments conducted on different days.

a

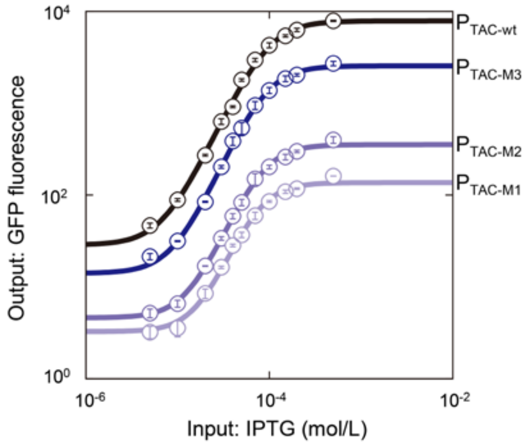

b

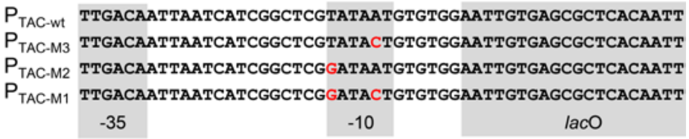

c

$$Output = \frac{\alpha\left(1+\left(\frac{Input_I}{K_I}\right)^{n_I}\right)}{1+\left(\frac{Input_I}{K_I}\right)^{n_I}+K_{lac}}+\beta$$

d

Parameters of P_{TAC} on P15A-AmpR

	α	β	K_I	n_I	K_{lac}
P_{TAC}	8,025	30.6			
$P_{TAC\text{-}M3}$	2,545	11.7	1.2×10^{-6}	2.2	14,088
$P_{TAC\text{-}M2}$	402	3.2			
$P_{TAC\text{-}M1}$	150	3.1			

Parameters of P_{TAC} on Chromosome

	α	β	K_I	n_I	K_{lac}
P_{TAC}	6,223	12.8			
$P_{TAC\text{-}M3}$	1,929	3.1	1.4×10^{-6}	2.3	15,719
$P_{TAC\text{-}M2}$	281	0.57			
$P_{TAC\text{-}M1}$	113	0.22			

Supplementary Figure 7. Parameterization of P_{TAC} Promoter Variants. (**a**) Response functions of P_{TAC} promoter variants using IPTG concentrations as the input. Open circles represent experimental data. The solid curves represent the fitting results according to the biophysical model in (c). Data were obtained by measuring the flow cytometric fluorescence of cells harboring pRG-sfGFP under the control of wild-type and mutant P_{TAC} promoters. Error bars represent the standard deviations from at least three biological replicates. (**b**) Sequence alignment of P_{TAC} promoter variants. Mutations are marked in red. (**c**) The biophysical model used to fit the response functions of P_{TAC} promoter variants. K_I and n_I represent the dissociation constants for IPTG and LacI, respectively; α and β denote the maximal and basal promoter activity. respectively; K_{lac} is the constant for LacI binding to lacO. (**d**) Parameter database of P_{TAC} promoters. The output of a P_{TAC} promoter carried on p15A-AmpR (pRG plasmid) is the input of transcriptional repression and the output of a P_{TAC} promoter on the chromosome is the input of transcriptional activation.

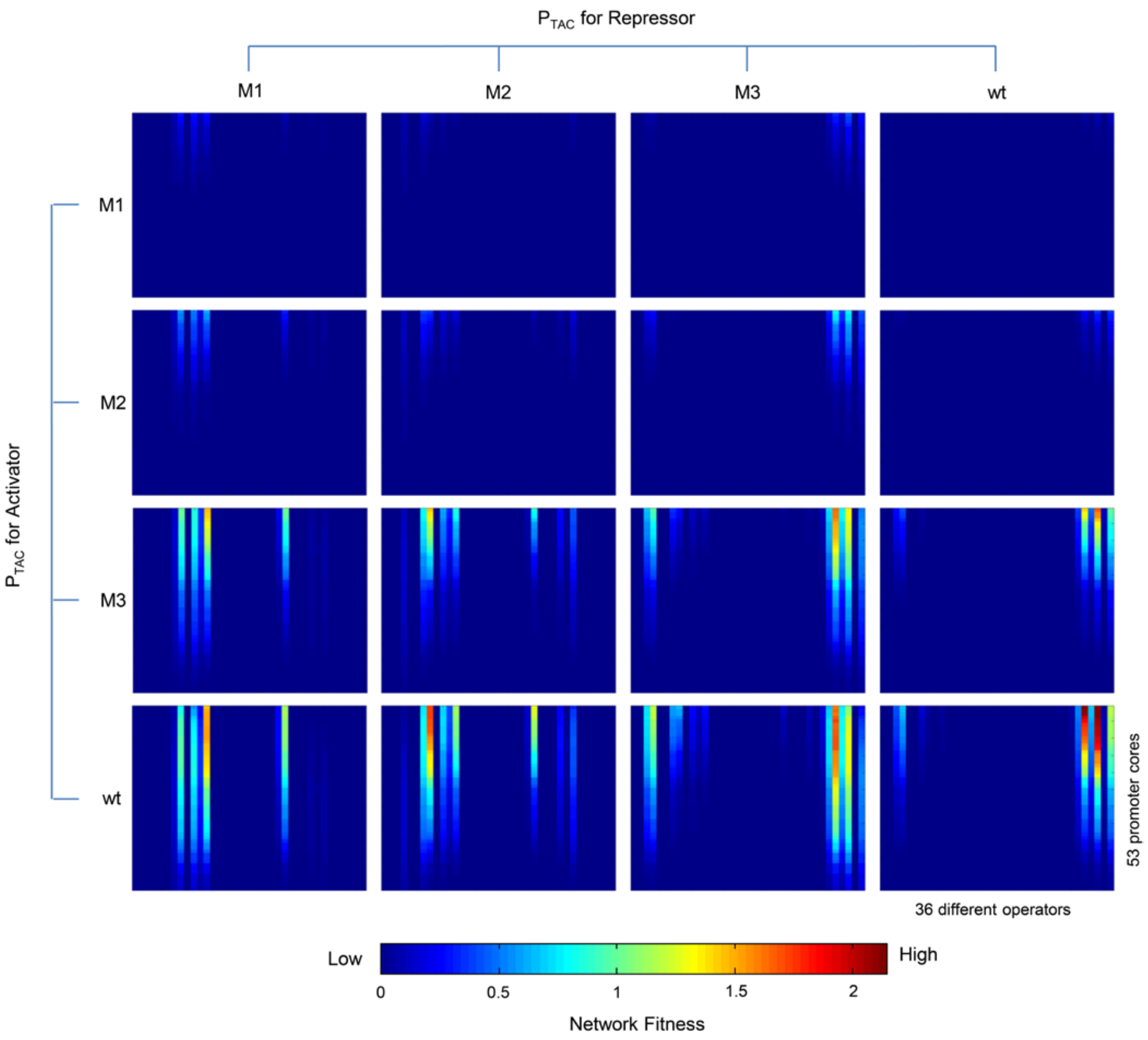

Supplementary Figure 8. Detailed Fitness Landscape of all 30,528 Network Designs. P_{TAC} for repressors: the promoter controlling the expression of repressors; P_{TAC} for activator: the promoter controlling the expression of T7 RNAP.

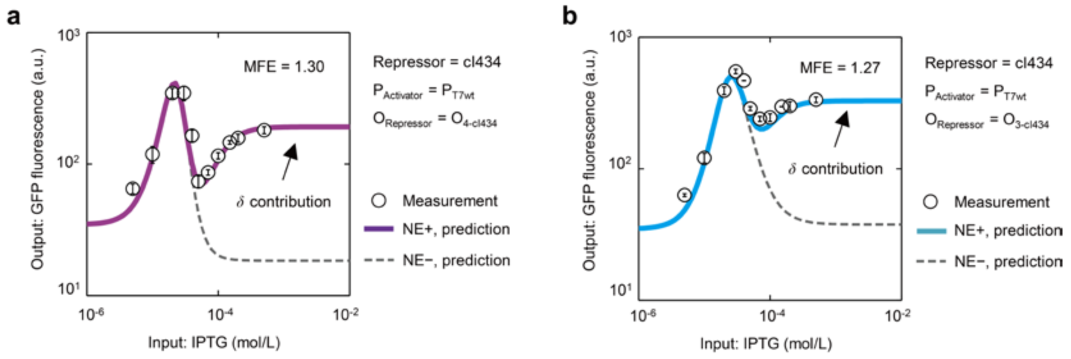

Supplementary Figure 9. **Experimentally determined and predicted response functions of IFFL networks using cI434 as the repressor.** (**a**) IFFL network based on the operator $O_{4\text{-cI434}}$. (**b**) IFFL network based on the operator $O_{3\text{-cI434}}$. NE+, with non-equilibrium correction term; NE-, without non-equilibrium term. MFE, mean relative (-fold) error. A secondary increase of GFP expression attributed to the non-equilibrium term is indicated. The output, GFP fluorescence, was measured using flow cytometry and quantified as the arithmetic means of measurements. Data represent the means ± SD from triplicate experiments conducted on different days.

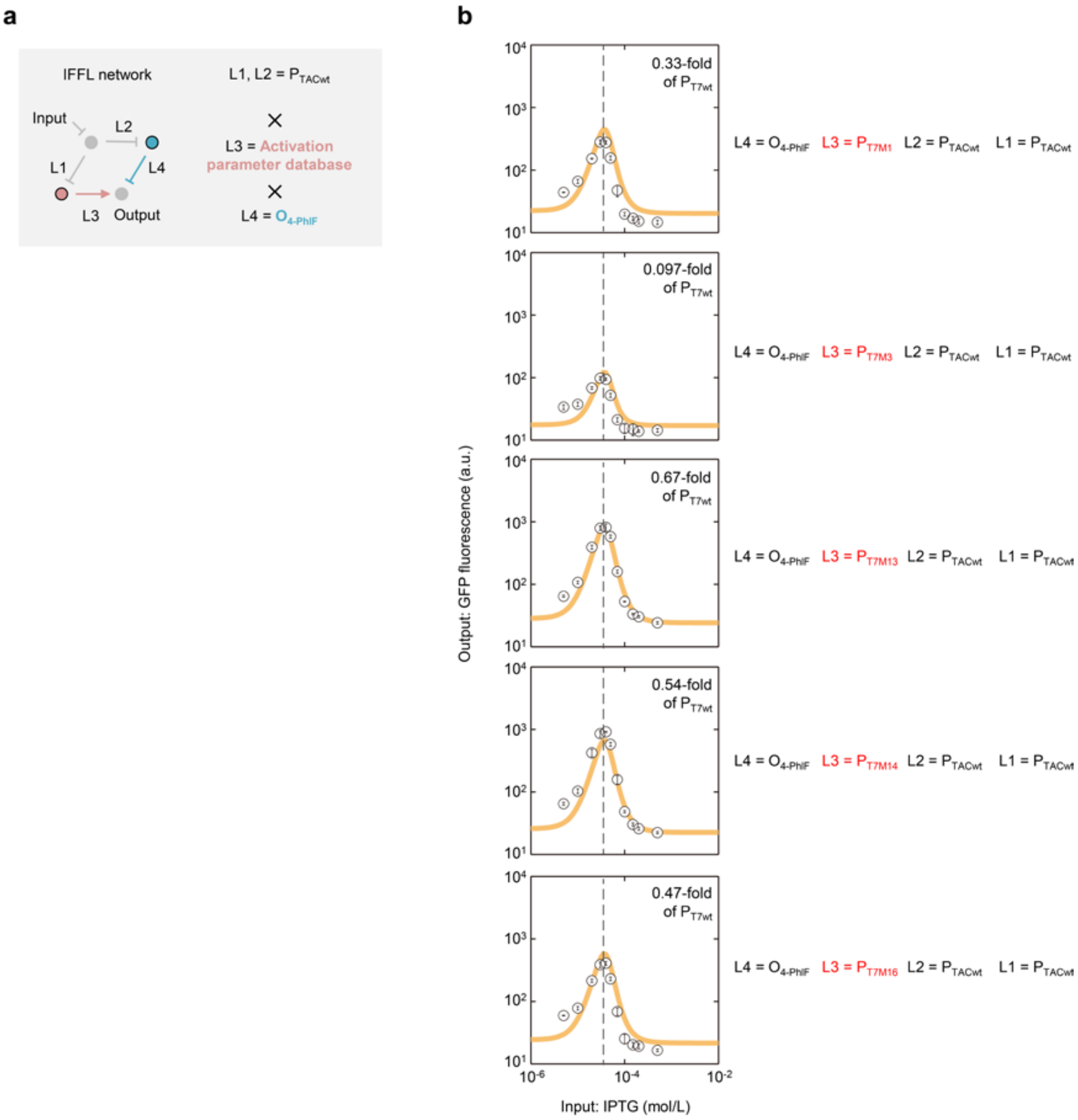

Supplementary Figure 10. Experimental and predicted response functions of re-designed IFFL networks. The wild-type T7 promoter of the highest-fitness IFFL network was replaced by five new promoter cores**.** (**a**) Schematic representation of network link assignment, design space and computation task. (**b**) Experimental and predicted response functions of IFFL networks using P_{T7M1}, P_{T7M3}, P_{T7M13}, P_{T7M14} and P_{T7M16} as the promoter cores instead of P_{T7wt}. The dashed line indicates the peak position of the highest-fitness IFFL circuit in Fig. 6f. Data represent the means ± SD from three replicate experiments conducted on different days.

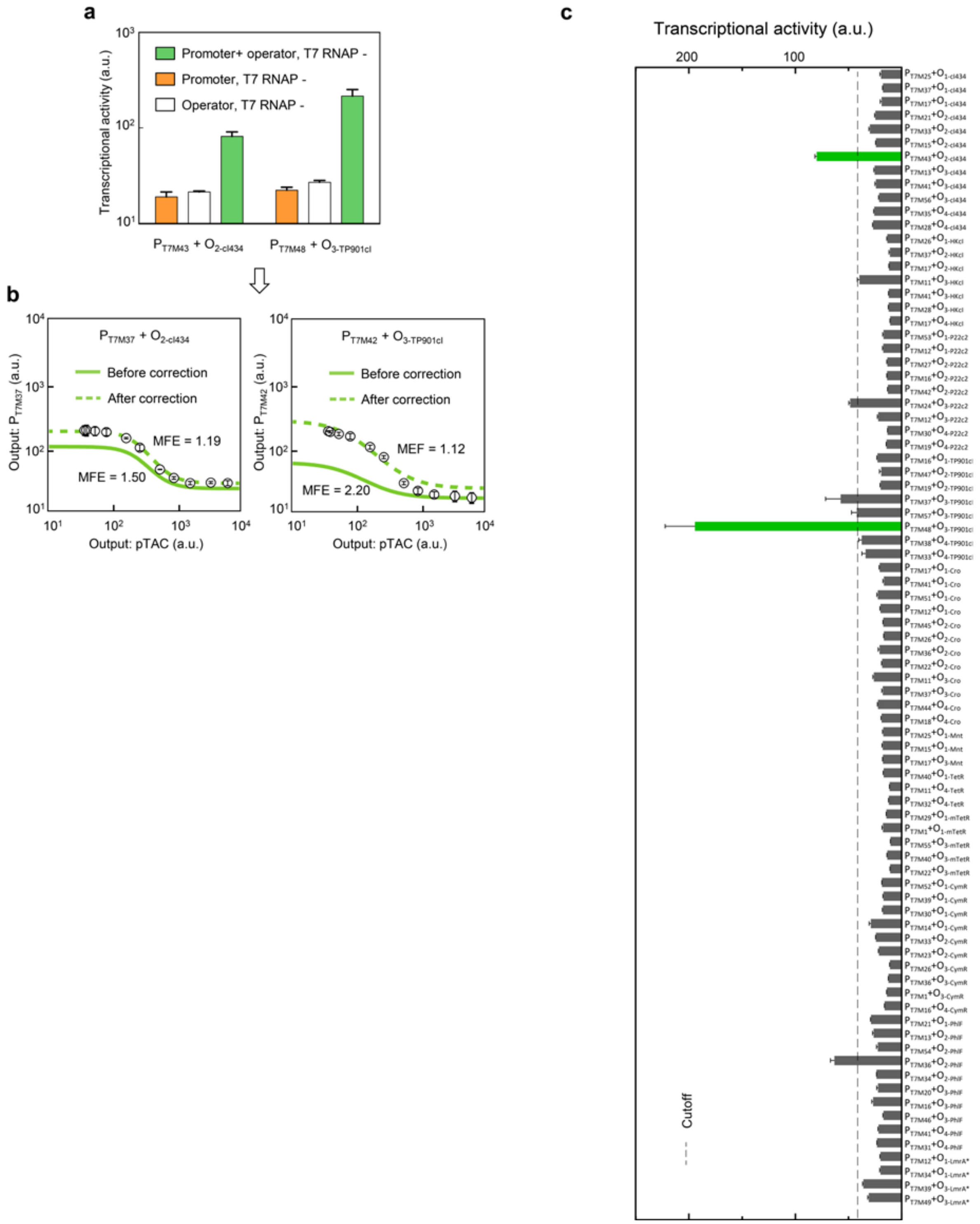

Supplementary Figure 11. Emergent Transcriptional Activity in Two Failed Combinatorial Promoter Designs. (**a**) Spontaneous (emergent) transcriptional activity of promoter cores, operators and their combinations in the absence of T7 RNAP. (**b**) Experimental and predicted response functions for two failed promoter designs before and after integrating the contribution of emergent promoter activity. MEF was calculated as described in Fig. 4. The promoter activity was calculated as the arithmetic mean of flow cytometry fluorescence data using sfGFP as the reporter. (**c**) Spontaneous Transcriptional Activity of all 83 Combinational Promoters in the Absence of T7 RNAP. The two combinational promoters shown in (b) are highlighted in green. The promoter activity was measured using superfolder GFP as the reporter and quantified as the arithmetic mean of flow cytometry fluorescence data. Data represent the means ± SD from at least three replicate experiments conducted on different days.

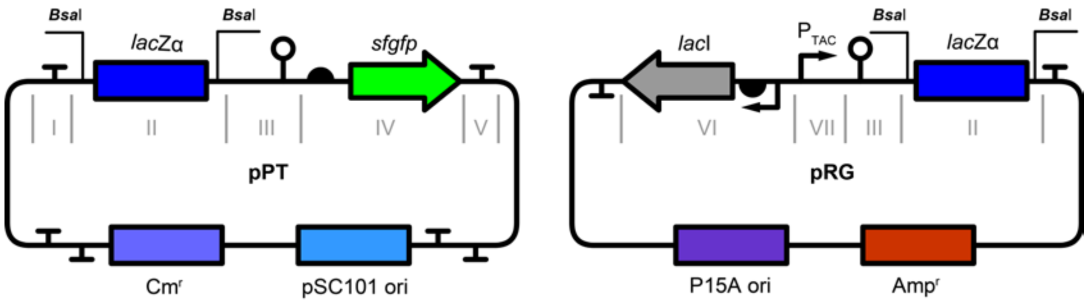

Supplementary Figure 12. Plasmid Architecture of pPT and pRG. The vast majority of the plasmids used in this study were derived from two basic vectors: pPT and pRG. We named the derived plasmids according to the schemes “pPT-XYZ” and “pRG-XYZ”, whereby XYZ denotes the sequence of interest by which the *lac*Zα fragment was replaced via Golden Gate Assembly. The crucial parts of the plasmid backbones are labeled with Roman numerals and identical parts are labeled with the same number. The corresponding sequences are summarized in Supplementary Table 4.

Supplementary Table 1. Sequences of Promoters, Single Operators, RNAPs, ECF σs and Transcriptional Repressors.

Name	Promoter[a]/Operator	RNAP/ECF σ/ Transcriptional Repressor[b]
T7	TAATACGACTCACTATA<br>GGGG	taccatagtactagagtcacacaggaaagtactagATGAACACGATTAACATCGCTAAGAACG<br>ACTTCTCTGACATCGAACTGGCTGCTATCCCGTTCAACACTCTGGCTGACCATTACGGTGAGC<br>GTTTAGCTCGCGAACAGTTGGCCCTTGAGCATGAGTCTTACGAGATGGGTGAAGCACGCTTCC<br>GCAAGATGTTTGAGCGTCAACTTAAAGCTGGTGAGGTTGCGGATAACGCTGCCGCCAAGCCTC<br>TCATCACTACCCTACTCCCTAAGATGATTGCACGCATCAACGACTGGTTTGAGGAAGTGAAAG<br>CTAAGCGCGGCAAGCGCCCGACAGCCTTCCAGTTCCTGCAAGAAATCAAGCCGGAAGCCGTAG<br>CGTACATCACCATTAAGACCACTCTGGCTTGCCTAACCAGTGCTGACAATACAACCGTTCAGG<br>CTGTAGCAAGCGCAATCGGTCGGGCCATTGAGGACGAGGCTCGCTTCGGTCGTATCCGTGACC<br>TTGAAGCTAAGCACTTCAAGAAAAACGTTGAGGAACAACTCAACAAGCGCGTAGGGCACGTCT<br>ACAAGAAAGCATTTATGCAAGTTGTCGAGGCTGACATGCTCTCTAAGGGTCTACTCGGTGGCG<br>AGGCGTGGTCTTCGTGGCATAAGGAAGACTCTATTCATGTAGGAGTACGCTGCATCGAGATGC<br>TCATTGAGTCAACCGGAATGGTTAGCTTACACCGCCAAAATGCTGGCGTAGTAGGTCAAGACT<br>CTGAGACTATCGAACTCGCACCTGAATACGCTGAGGCTATCGCAACCCGTGCAGGTGCGCTGG<br>CTGGCATCTCTCCGATGTTCCAACCTTGCGTAGTTCCTCCTAAGCCGTGGACTGGCATTACTG<br>GTGGTGGCTATTGGGCTAACGGTCGTCGTCCTCTGGCGCTGGTGCGTACTCACAGTAAGAAAG<br>CACTGATGCGCTACGAAGACGTTTACATGCCTGAGGTGTACAAAGCGATTAACATTGCGCAAA<br>ACACCGCATGGAAAATCAACAAGAAAGTCCTAGCGGTCGCCAACGTAATCACCAAGTGGAAGC<br>ATTGTCCGGTCGAGGACATCCCTGCGATTGAGCGTGAAGAACTCCCGATGAAACCGGAAGACA<br>TCGACATGAATCCTGAGGCTCTCACCGCGTGGAAACGTGCTGCCGCTGCTGTGTACCGCAAGG<br>ACAAGGCTCGCAAGTCTCGCCGTATCAGCCTTGAGTTCATGCTTGAGCAAGCCAATAAGTTTG<br>CTAACCATAAGGCCATCTGGTTCCCTTACAACATGGACTGGCGCGGTCGTGTTTACGCTGTGT<br>CAATGTTCAACCCGCAAGGTAACGATATGACCAAAGGACTGCTTACGCTGGCGAAAGGTAAAC<br>CAATCGGTAAGGAAGGTTACTACTGGCTGAAAATCCACGGTGCAAACTGTGCGGGTGTCGATA<br>AGGTTCCGTTCCCTGAGCGCATCAAGTTCATTGAGGAAAACCACGAGAACATCATGGCTTGCG<br>CTAAGTCTCCACTGGAGAACACTTGGTGGGCTGAGCAAGATTCTCCGTTCTGCTTCCTTGCGT<br>TCTGCTTTGAGTACGCTGGGGTACAGCACCACGGCCTGAGCTATAACTGCTCCCTTCCGCTGG<br>CGTTTGACGGGTCTTGCTCTGGCATCCAGCACTTCTCCGCGATGCTCCGAGATGAGGTAGGTG<br>GTCGCGCGGTTAACTTGCTTCCTAGTGAAACCGTTCAGGACATCTACGGGATTGTTGCTAAGA<br>AAGTCAACGAGATTCTACAAGCAGACGCAATCAATGGGACCGATAACGAAGTAGTTACCGTGA<br>CCGATGAGAACACTGGTGAAATCTCTGAGAAAGTCAAGCTGGGCACTAAGGCACTGGCTGGTC<br>AATGGCTGGCTTACGGTGTTACTCGCAGTGTGACTAAGCGTTCAGTCATGACGCTGGCTTACG<br>GGTCCAAAGAGTTCGGCTTCCGTCAACAAGTGCTGGAAGATACCATTCAGCCAGCTATTGATT<br>CCGGCAAGGGTCTGATGTTCACTCAGCCGAATCAGGCTGCTGGATACATGGCTAAGCTGATTT<br>GGGAATCTGTGAGCGTGACGGTGGTAGCTGCGGTTGAAGCAATGAACTGGCTTAAGTCTGCTG<br>CTAAGCTGCTGGCTGCTGAGGTCAAAGATAAGAAGACTGGAGAGATTCTTCGCAAGCGTTGCG<br>CTGTGCATTGGGTAACTCCTGATGGTTTCCCTGTGTGGCAGGAATACAAGAAGCCTATTCAGA<br>CGCGCTTGAACCTGATGTTCCTCGGTCAGTTCCGCTTACAGCCTACCATTAACACCAACAAAG<br>ATAGCGAGATTGATGCACACAAACAGGAGTCTGGTATCGCTCCTAACTTTGTACACAGCCAAG<br>ACGGTAGCCACCTTCGTAAGACTGTAGTGTGGGCACACGAGAAGTACGGAATCGAATCTTTTG<br>CACTGATTCACGACTCCTTCGGTACCATTCCGGCTGACGCTGCGAACCTGTTCAAAGCAGTGC<br>GCGAAACTATGGTTGACACATATGAGTCTTGTGATGTACTGGCTGATTTCTACGACCAGTTCG<br>CTGACCAGTTGCACGAGTCTCAATTGGACAAAATGCCAGCACTTCCGGCTAAAGGTAACTTGA<br>ACCTCCGTGACATCTTAGAGTCGGACTTCGCGTTCGCGTAA
σ^{ECF11}_{1}	TGATCCACTCTTCATCC<br>CGCTACGTAACACCTCT<br>GCATCGCGAACCAAAAC	caaaaccagagctactagATGATGAGCGATAGTCCGCAGAAACTGGGTCGTAATGAATGGAAT<br>GCCTATATGGATAAAGTGAAAGCCAAAGATCGTGAAGCCTTTGCATTTGTGTTTCGTTTTTAT<br>GCACCGAAACTGAAACAGTTCGCCTATAAACATGTGGGTAATGAACAGGTTGCCATGGAAATG

	C	GTTCAAGAAACCATGGCCACCGTTTGGCAGAAAGCACATCTGTATGATGGTAAAAAAAGCGCA<br>CTGAGCACCTGGATTTATACCATTATTCGCAACCTGTGCTTTGATCTGCTGCGTAAACAGAAA<br>GGTAAAGAACTGCATATCCACTCCGATGATATTTGGCCGAGCGAATATTATCCGCCTGATATG<br>GTTGATCACTATAGTCCGGAACAGGATATGCTGAAAGAACAGGTGGTGAAATTTCTGGATATC<br>CTGCCGAAAAATCAGCGTGATGTTCTGCAGGCAGTTTATCTGGAAGAACTGCCGCATCAGCAG<br>GTTGCAGAACTGTTTGATATTCCGCTGGGCACCGTTAAAAGCCGTCTGCGTCTGGCAGTTGAA<br>AAACTGCGTCATAGCATGCATACCGAACAGCTGTAA
gh-1	TTAAAAACCCTCACTATGGC	tatccaaaccagtagctcaattggagtcgtctatATGACCATCGCTATCCCGGAACGTCACGA<br>CTTCTCTGACATCAACTCTTCTGCTGCTTTCGACGCTCTGTCTAACATCTACGGTCCGGCTCT<br>GGCTGCTGAACAGCTCCAGCTGGAACACGAAGCTTACACCCTGGGTGAAGAACGTTTCCACAA<br>AGCTATGGAACGTCAGATGGAACGTGGTGAGTTCTCTAACTCTCAGGTTGCTAAACCGCTGCT<br>GGGTCACCTGGTTCCGATGCTGTCTAAAGCTATCACCGACTGGATCGAACACCAGACCACCAA<br>AGTTCGTCGTAAACACGTTGCTCTGGGTGCTTTCCAGCAGATGAACCCGGAAACCATGGCTTC<br>TATCGTTATCCGTTGGACCATCAACCGTATCGCTCAGCGTTCTGGTGCTCCGACCATCACCGA<br>AATGGCTGTTTCTATCGGTGGTGCTCTGGAAGAAGAAGCTCGTTTCGGTCGTATCCGTGTTCT<br>GGAACAGCAGCACTACCAGAAACACATCAAAAAAGCTCTGGCTCAGCGTAACGGTATGACCTA<br>CAAAGTTGCTTACATGGAAAAAGTTGAAGCTCACATGATCGAAGCTGGTCAGCTGAACGAACC<br>GTGGACCGAATGGGACCAGTCTGGTGCTGACGTTCGTTACCACATGGGTATCCGTATGCTGGA<br>ACTGCTGATCGAATCTACCCAGCTGATCGAAGTTGTTCGTGAACACAAAGGTAACAAAAAACT<br>GGACGGTGAATACGTTTACCTGAAAGCTGAATGGGCTGACAAACTCCAGTCTCGTGCTTACAT<br>CCTGTCTGGTGTTTTCCCGCGTTACCAGCCGATGGTTGTTCCGCCGAAACCGTGGAACGGTGT<br>TCGTGGTGGTGGTTACTGGGCTAAAGGTCGTAAACCGGTTACCTTCATCCGTGTTCCGACCAA<br>ACGTGCTCTGAACCGTTACCGTGACGTTCACATGCCGGAAGTTTACAAAGCTGTTAACCTGGC<br>TCAGGCTACCCCGTGGGCTATCAACCAGAAAGTTCTGGCTGTTGCTAACGCTGTTATGTCTTG<br>GGAAAACGTTCCGATCAAAGAGTTCCCGTCTACCGAACGTGAAGCTCTGCCGATCAAACCGGG<br>TGACATCGAAACCAACGAAGAAGCTCTGAAAGCTTGGAAAAAAGCTGCTGCTGGTGTTTACCG<br>TAAAGACGCTGCTCGTGTTTCTCGTCGTCTGTCTTACGAGTTCTCTCTGGAACAGGCTAACAA<br>ATTCGCTGAATACGACGCTATCTACTTCCCGTACAACCTGGACTGGCGTGGTCGTGTTTACGC<br>TATCCCGGCTTTCAACCCGCAGTCTAACGACATGACCAAAGGTATCCTCCAGGCTGCTAAAGG<br>TGAACCGGTTGGTAAAGACGGTATCGAATGGCTGATGATCCACGGTGCTAACTGCGCTGGTGT<br>TGACAAAGTTGACTTCTCTCAGCGTAAACAGTGGATCAAAGACAACGAAGAAATGATCCTGCG<br>TTGCGCTCACGACCCGCTGATCAACACCGACTGGATGGACATGGACTCTCCGTTCTGCTTCCT<br>GGCTTTCTGCTTCGAATGGCAGGGTGTTAAACTGCACGGTGAAGCTCACGTTTCTGCTCTGCC<br>GATCGCTTTCGACGGTTCTTGCTCTGGTATCCAGCACTTCTCTGCTATGCTGCGTGACGAACG<br>TGGTGGTCGTGCTGTTAACCTGCTCCAGTCTGACGACGTTCAGGACATCTACAAACTGGTTTC<br>TGACGAAGTTGAAATCGCTCTCCAGTGGGACCTGAAATACGGTACCGAGGACTCTACCGTTCT<br>GGACACCAACGAGGACACCGGTGAAATCACCGAACGTCGTGTTCTGGGTACCAAAACCCTGGC<br>TATGGCTTGGCTGACCTACGGTATGTCTCGTAAAGTTACCAAACGTTCTGTTATGACCCTGGC<br>TTACGGTTCTAAAGCTTACGGTTTCGCTGACCAGGTTCGTGAGGACATCGTTAAAAAAGCTAT<br>CGACAACGGTGACGGTGAAATGTTCACCTCTCCGGGTGAAGCTTCTCGTTACATGGCTGGTAA<br>AATCTGGGACTCTGTTTCTGTTGTTGTTGTTGCTGCTGTTGAAGCTATGAACTGGCTCCAGAA<br>AGCTGCTAAACTGCTGGCTTCTGAAGTTAAATGCAAAAAAACCAAACAGGTTCTGAAACCGGC<br>TATGCCGGTTTACTGGGTTACCCCGGACGGTTTCCCGGTTTGGCAGGAATACATGATCCCGGA<br>AACCCGTCGTATCGACCTGATGTTCCTGGGTGACGTTCGTATCCAGGCTACCGTTACCGTTCG<br>TGACTCTGACAAAATCGACGCTCGTAAACAGGAATCTGGTATCTCTCCGAACTTCGTTCACTC<br>TCAGGACGGTTCTCACCTGCGTAAAACCGTTGTTCACGCTGCTGAACGTTACGGTATCGAGTT<br>CTTCGCTCTGATCCACGACTCTTTCGGTACCATCCCGGCTCACGCTGGTGCTATGTTCAAAGC<br>TGTTCGTGAAACCATGGTTGAAACCTACGAATCTAACAACGTTCTGGAGGACTTCCGTGAACA

		GTTCATGGACCAGCTGCACGAATCTCAGCTGGACAAAATGCCGCCGATCCCGGAAATGGGTAC<br>CCTGGACATCCGTGAAATCCTGAAATCTCAGTTCGCTTTCGCTTAA
MmP-1	GTGTGGCGAGCAGTGTA<br>GGACATATTTGTGGCAT<br>TATAGGGAG	tatccaaaccagtagctcaattggagtcgtctatATGTCAATTGCGGCGGCGGTGAACAAAAA<br>TGATTTCTCGGACGTTGAACTGGCTGCGATCCCGTTTAACACCCTGGCGGACCATTACGGTGC<br>GGATCTGGCCCGTGAACAGCTGCAACTGGAACACGAAAGCTATGTGATGGGCGAAGAACGTTT<br>CCGCAAAATGCTGGAACGCCAGGAAAAAGCGGAAGAATTTGGTGATAGCTCTGTTGCCAAACC<br>GCTGATTATCACGCTGCTGCCGAAAGTCACGCAGCGTATTACCGACTGGCTGAACGAATGGGC<br>AGATCCGAATAAAAAAGGCCGCAAACCGATTGCTTATACCCATCTGAAAGATATCAAACCGGA<br>AACGCTGGCCTTCATTACCATCAAAGTGGTTCTGAATAAACTGGCGGGTAAAGATGACGCCTT<br>TATGCAGCCGCTGGCATACGCTATTGGTAGTTCCATCGAAGATGAAGCACGTTTCGGCCGTAT<br>CCGCGAACTGGAAATGGCACACTTTAAAAAATGCGCTGAAGAAAACCTGAATAAACGTCGCGG<br>CACCGCGTATCGCAAAGCCTTTCTGAGTGTCGTGGAAGCGGATATGCTGGACAAAGGTCTGCT<br>GGGCGGTGAATCATGGGGCACGTGGAACAAAACCGATGTGATGAATATTGGTATCTCGATGCT<br>GGAAAAACTGATTGAAGCCACGGGCCTGGTTGAACTGCGTGAAAAACGCAACTTTGAAGAAAT<br>GGATCGTATTGTCATCGCAGAAGAATACGTGAAAGCGATGGCCACCCGCGCACAGTCACTGGC<br>TGGCATCTCGCCGATGTATCAACCGTGTGTTGTCCCGCCGAAACCGTGGGTGAGCATTACGGG<br>CGGTGGCTACTGGGCAAACGGTCGTAAACCGACCGCTCTGATCCGTACCCATACGCGCAAAGC<br>ACTGTATCGCTACGAAGATGTTTATATGCCGGAAGTCTACAAAGCGATTAATTATGCCCAGGA<br>AACCCCGTGGCGTATCAACCGCAAAGTGCTGGCGGTGGTTAACGAACTGGTTAAATGGAAAAA<br>CAACCCGGTCAAAGACATGCCGAGCATTGATAAACTGGAACTGCCGCAGCGTCCGGATGACAT<br>CGATACCAACGAAGAAGCGCTGCGTTCTTGGAAACGCGAAGCCGCAGCTGTTTACCGCAAAGA<br>TGAACAGCGTAAAAGCCGCTATCTGAGTATGTCCTTTGCACTGGAACAAGCTAACAAATTCTC<br>TAACAAAAAAGCAATCTACTTCCCGTACAATATGGACTGGCGTGGCCGCGTCTATGCACTGCC<br>GATGTTCAACCCGCAGGGTAATGATATGGTTAAAGGCCTGCTGACCCTGGCCAAAGGTAAACC<br>GATTGGTAAAGACGGCTTTTACTGGCTGAAAATCCATGGTGCAAACACGGCTGGCGTCGATAA<br>AGTGACCTTTCCGGAACGTATTAAATTCATCGAAGATAACCACGACAATATTATGCAGTGCGC<br>GGAAAGCCCGCTGGACAATCTGTGGTGGACGGAACAAGATTCTCCGTTTTGCTTCCTGGCGTT<br>TTGTTTCGAATATGCCCAGGTCACCAAAAAAGGTCTGGGCTGGGTGTGCAGTCTGCCGATTGC<br>CCTGGATGGTTCATGTTCGGGCATCCAACACTTTTCCGCAATGCTGCGTGATGACATTGGTGG<br>CCGCGCTGTTAACCTGCTGCCGAGCGAAACCGTCCAGGACATTTATGGTATCGTGGCAGATAA<br>AGTTAATGAAGCTCTGAAAGAACTGGTCATCAACGGCACGGATAATTACACCGACACGGTGAC<br>CGATAAATCTACCGGTGAAATTATCGAACGTTATCGCCTGGGCGAAAAAGAACTGGCGCGTCA<br>GTGGCTGGAATTTGGCGTCACGCGTAGCGTGACCAAACGCTCTGTGATGACCCTGGCCTACGG<br>TTCAAAAGAATATGGCTTTCGTGACCAGGTTCTGGAAGATACGATTCGCCCGGCGATCGATTC<br>GGGTAAAGGCGCCATGTTCACCAATCCGAGTCAAGCGGCCTCCTTTATGGCGAAACGCATTTG<br>GGAAGCCGTGAGCGTTACCGTCGTGGCAGCTGTGGGTGCGATGAAATGGCTGCAATCATCGGC<br>CAAACTGATGGCGGCCGAAGTGAAAGACAAGAAAACCAAAGAAGTTCTGCGTAAACGCTGCGC<br>GGTTCATTGGGTCACCCCGGATGGTTTCCCGGTGTGGCAGGAATATCGTAAACCGAAACAAAA<br>ACGCGTTCACCTGATGTTTCTGGGTAGTTATTACGATGCGCGTATGAAAGAAACGAGCTCTGA<br>CTGTTCCATTGATGCCCATAAACAGGAAAGCGGTATCTCTCCGAACTTCGTGCATAGCCAAGA<br>TGGCAATCACCTGCGTATGACCGTTGTCTACGCGCGCGAAAAATATAACGTGGAAAGTTTTGC<br>CCTGATTCACGACTCCTTCGGCACGATCCCGGCAGATGTTCCGAACCTGTTTAAAGCTGTGCG<br>CGAAACCATGGTTAATATGTACGAAAACAATGACGTGCTGGCAGATTTTTATGAACAGTTCGC<br>TGACCAACTGCATGAAAGTCAGCTGGATAAAATGCCGGCGCTGCCGCCGAAAGGTAAACTGAA<br>TCTGCAAGACATTCTGAAATCCGATTTTGCATTCGCTTAA
T3	ATTAACCCTCACTAAAG<br>GGAA	tactagagtatcagaactagatactagATGAACATCATCGAAAACATCGAAAAGAATGACTTC<br>TCAGAAATCGAACTGGCTGCTATCCCGTTCAACACACTGGCTGACCACTACGGAAGCGCCTTG<br>GCTAAAGAGCAGTTGGCTTTAGAACATGAGTCTTATGAGCTAGGCGAGCGCCGCTTCCTCAAG

		ATGCTTGAGCGTCAAGCGAAAGCTGGTGAGATTGCAGACAACGCAGCCGCTAAGCCGTTACTC<br>GCTACGCTTCTCCCTAAGTTAACCACACGTATCGTCGAGTGGCTCGAAGAGTACGCATCGAAG<br>AAAGGCCGCAAGCCTAGCGCATACGCACCGCTCCAGTTACTCAAGCCGGAGGCCTCCGCGTTT<br>ATCACCCTGAAAGTTATCCTTGCGTCACTAACCAGTACGAACATGACAACCATTCAGGCCGCT<br>GCTGGTATGCTGGGGAAAGCCATTGAGGACGAGGCACGATTTGGGCGCATCCGTGACCTAGAA<br>GCGAAGCACTTCAAGAAGCACGTTGAGGAACAGCTTAACAAGCGCCACGGGCAAGTCTACAAG<br>AAAGCATTTATGCAGGTGGTCGAGGCCGATATGATTGGTCGAGGTCTGCTTGGTGGCGAGGCG<br>TGGTCTAGCTGGGATAAAGAAACCACGATGCACGTAGGGATTCGCCTGATTGAAATGCTGATT<br>GAATCCACGGGTCTGGTGGAATTACAGCGCCACAACGCAGGTAACGCAGGCTCTGACCATGAG<br>GCACTGCAACTGGCCCAAGAGTACGTGGACGTATTAGCGAAGCGTGCAGGCGCTCTGGCGGGT<br>ATCTCTCCGATGTTCCAGCCGTGTGTCGTACCGCCGAAACCTTGGGTAGCAATCACAGGGGGC<br>GGCTATTGGGCTAACGGTCGCAGACCTTTGGCACTCGTTCGCACTCACTCTAAGAAGGGCTTG<br>ATGCGCTACGAAGACGTTTACATGCCAGAAGTCTACAAGGCTGTGAACCTCGCGCAAAACACC<br>GCATGGAAAATCAACAAGAAAGTTCTTGCTGTTGTCAATGAGATTGTTAACTGGAAGAATTGC<br>CCGGTAGCAGACATTCCATCGCTGGAGCGCCAAGAGTTACCGCCTAAGCCTGACGACATTGAC<br>ACCAACGAGGCAGCGCTCAAGGAGTGGAAGAAAGCCGCTGCTGGTATCTATCGCTTGGACAAG<br>GCACGAGTGTCTCGCCGTATCAGCTTAGAGTTCATGCTGGAGCAGGCCAACAAGTTCGCAAGT<br>AAGAAAGCAATCTGGTTCCCTTACAACATGGACTGGCGCGGTCGTGTGTACGCTGTGCCGATG<br>TTCAACCCGCAAGGCAACGACATGACGAAAGGTCTGCTGACCCTTGCTAAAGGCAAGCCAATC<br>GGTGAGGAAGGTTTCTACTGGCTGAAAATCCACGGTGCGAACTGTGCGGGTGTTGATAAGGTT<br>CCATTCCCGGAGCGCATCGCGTTCATTGAGAAGCACGTAGACGACATTCTGGCTTGCGCTAAA<br>GACCCAATCAATAACACTTGGTGGGCTGAGCAGGATTCACCGTTCTGTTTCCTCGCGTTTTGC<br>TTCGAGTATGCAGGCGTTACGCACCACGGTCTGAGCTACAATTGCTCTCTGCCGCTGGCGTTC<br>GACGGGTCTTGCTCTGGTATCCAGCACTTCTCCGCGATGCTCCGCGATGAGGTAGGCGGTCGT<br>GCGGTTAACCTGCTGCCAAGCGAAACCGTGCAGGACATTTACGGCATCGTTGCACAGAAAGTA<br>AACGAGATTCTCAAACAGGATGCAATCAACGGCACGCCTAACGAGATGATTACCGTGACCGAC<br>AAGGACACCGGGGAAATCTCAGAGAAGCTCAAACTTGGAACCTCAACGCTGGCGCAACAGTGG<br>CTGGCATATGGTGTAACCCGTAGCGTAACTAAACGTTCGGTCATGACGCTGGCTTACGGTTCC<br>AAGGAGTTCGGCTTTCGTCAACAGGTATTGGATGACACCATTCAGCCTGCAATTGACAGCGGT<br>AAGGGCTTGATGTTCACCCAACCGAACCAAGCGGCTGGCTATATGGCTAAGCTGATTTGGGAT<br>GCGGTAAGCGTGACCGTAGTTGCAGCGGTTGAGGCGATGAACTGGCTCAAATCTGCCGCTAAG<br>CTGCTGGCTGCTGAGGTCAAGGACAAGAAGACCAAGGAGATTCTGCGCCACCGTTGCGCGGTT<br>CACTGGACTACGCCGGACGGCTTCCCGGTCTGGCAGGAATACCGCAAGCCACTCCAGAAGCGT<br>CTCGATATGATTTTCTTAGGGCAATTCCGTCTGCAACCGACGATTAATACCCTCAAGGATTCA<br>GGCATTGACGCACACAAGCAGGAGTCTGGCATCGCTCCTAACTTTGTTCACTCACAGGACGGT<br>AGCCACCTCCGCATGACAGTCGTTTATGCTCACGAGAAGTATGGCATTGAGTCCTTTGCGCTC<br>ATCCATGACAGCTTTGGGACTATCCCGGCAGACGCTGGTAAGCTCTTTAAGGCTGTGCGTGAA<br>ACGATGGTTATCACCTATGAGAACAACGATGTGCTGGCAGACTTCTACTCTCAGTTTGCCGAC<br>CAGCTACACGAGACCCAACTGGACAAGATGCCTCCGCTTCCGAAGAAAGGAAACCTGAACCTG<br>CAAGACATTCTCAAGTCTGACTTTGCCTTTGCATAA
σ^{ECF16}<br>1	GTAACCCTGGCGGCCGA<br>TGCAACGAACTAACTCA<br>CAGGACGTGCTCAGCAC<br>C	tcacacaggaaaggcctcgATGCAGCGTACCAACTCTCAGGACGAACTGGAAGCTCGTGAAGC<br>TCGTCTGAAAGCTCTGCTGCTCCAGGGTCTGACCGGTGACCAGCTGGCTTACCAGGACTTCCT<br>GGGTACCCTGGCTCTGCACGTTCGTGCTTTCCTGCGTTCTCGTCTGTCTCGTCGTCCGGCTGA<br>AATCGAAGACATGGTTCAGGACGTTCTGCTGGCTGTTCACAACGCTCGTCACACCTACCAGCC<br>GCAGCAGCCGCTGACCGCTTGGGTTCAGGCTATCGCTCGTTACAAACTGGCTGACCACCTGCG<br>TTCTGTTTCTCGTCGTGACGCTCGTCACGACGTTCTGGACGACGACGCTCAGCTGTTCGCTGT<br>TTCTGAAGTTGAATCTGCTGAAGCTTCTCGTGACCTGACCAAACTGCTGAAACAGCTGCCGGA<br>ACGTCAGCGTCTGCCGATCGTTCACGTTAAACTGGAAGGTCTGTCTGTTGAAGAAACCGCTCA

		GCTGACCGGTCTGTCTTCTTCTGCTGTTAAAGTTGGTATCCACCGTGGTCTGAAAGCTCTGTCTCGTCTGATCGGTGGTGCTAACAACCACGAAGACTAA
σ^{ECF20}<br>1	ACGGATTCCCCGCCCATCTATCGTTGAACCCATCAGCTGCGTTCATCAGCGA	tcacacaggaaaggcctcgATGAACGAAACCGACCCGGACCTGGAACTGCTGAAACGTATCGGTAACAACGACGCTCAGGCTGTTAAAGAAATGGTTACCCGTAAACTGCCGCGTCTGCTGGCTCTGGCTTCTCGTCTGCTGGGTGACGCTGACGAAGCTCGTGACATCGCTCAGGAATCTTTCCTGCGTATCTGGAAACAGGCTGCTTCTTGGCGTTCTGAACAGGCTCGTTTCGACACCTGGCTGCACCGTGTTGCTCTGAACCTGTGCTACGACCGTCTGCGTCGTCGTAAAGAACACGTTCCGGTTGACTCTGAACACGCTTGCGAAGCTCTGGACACCCGTCCGGCTCCGGACGAACAGCTGGAGGCTTCTGCTCAGTCTCGTCGTATGGCTCAGGCTCTGGACCAGCTGCCGGACCGTCAGCGTGAAGCTATCGTTCTCCAGTACTACCAGGAACTGTCTAACACCGAAGCTGCTGCTCTGATGCAGATCTCTGTTGAAGCTCTGGAATCTCTGCTGTCTCGTGCTCGTCGTAACCTGCGTTCTCACCTGGCTGAAGCTCCGGGTGCTGACCTGTCTGGTCGTCGTAAACCGTAA
CI434<br>2	TACAAGAAAGTTTGTT	tactagagaaagaggagaaatactagATGAGTATTTCTTCCAGGGTAAAAAGCAAAAGAATCCAGCTTGGACTTAACCAGGCTGAACTTGCTCAAAAGGTGGGGACTACCCAGCAGTCTATAGAGCAGCTCGAAAACGGTAAAACTAAGCGACCACGCTTTTTACCAGAACTTGCGTCAGCTCTTGGCGTAAGTGTTGACTGGCTGCTCAATGGCACCTCTGATTCGAATGTTAGATTTGTTGGGCACGTTGAGCCCAAAGGGAAATATCCATTGATTAGCATGGTTAGAGCTGGTTCGTGGTGTGAAGCTTGTGAACCCTACGATATCAAGGACATTGATGAATGGTATGACAGTGACGTTAACTTATTAGGCAATGGATTCTGGCTGAAGGTTGAAGGTGATTCCATGACCTCACCTGTAGGTCAAAGCATCCCTGAAGGTCATATGGTGTTAGTAGATACTGGACGGGAGCCAGTGAATGGAAGCCTTGTTGTAGCCAAACTGACTGACGCGAACGAAGCAACATTCAAGAAACTGGTCATAGATGGCGGTCAGAAGTACCTGAAAGGCCTGAATCCTTCATGGCCTATGACTCCTATCAACGGAAACTGCAAGATTATCGGTGTTGTCGTGGAAGCGAGGGTAAAATTCGTATAG
HKCI<br>3	TGAACCATAAGTTCA	tactagaaagtaccgtcatactagATGGTTCAACAGAAAGAGCGTGAAACTTTCTCGCAGAGGCTTGCGCTGGCCTGTGATAAAGCGGGATTACCTTTGCATGGTAGGCAGGCTGATTTAGCTGTCAGGCTTAAGGTCACACCAAAAGCCATTAGTAAATGGTTCAACGGGGAGTCAATACCAAGAAAAGACAAGATGGAATCTCTGGCTTCGGTGCTGGGAACTACTGCTGCATATCTGCATGGCTATGCTGATGATGACGGTATCACGGTAAATCATCTATCAAGATCAAATGATTATTATCGTGTTGATGTATTGGATGTTCAGGCGAGCGCCGGGCCAGGAACCATGGTTTCCAATGAATTTATAGAAAAGATAAGAGCAATTGAATATACGACCGAGCAGGCAAGAATTTTATTTAATGGAAGGCCACAGGAAAGCGTAAAAGTCATCACGGTTCGCGGTGACAGCATGGAGGGAACCATCAATCCGGGAGATGAGATCTTTGTTGATGTATCCATAACCTGTTTTGATGGCGATGGCATTTATGTGTTTGTATACGGGAAAACAATGCACGTTAAGCGCCTGCAAATGCAAAAGAACAGGCTTGCCGTCATCTCTGACAATGCCGCTTATGATCGATGGTACATAGAAGAAGGTGAAGAAGAGCAACTTCACATTCTAGCCAAAGTCCTCATTAGGCAGTCAATCGATTACAAGCGATTCGGATGA
P22C2<br>4	ATTTAAGTGTTCTTTAAT	tactagaaagcactttaatactaGTGAATACACAATTGATGGGTGAGCGTATTCGCGCTCGAAGAAAAAAACTCAAGATTAGACAAGCCGCTCTTGGTAAGATGGTGGGAGTGTCTAATGTTGCAATATCGCAATGGGAGCGCTCGGAGACTGAGCCAAATGGGGAGAACCTGTTGGCACTTTCGAAGGCTCTTCAGTGCTCCCCTGACTATTTGCTGAAAGGAGATTTAAGCCAGACAAACGTTGCCTATCATAGTAGGCATGAGCCAAGAGGATCATACCCTCTTATCAGTTGGGTAAGCGCAGGGCAATGGATGGAAGCTGTAGAACCTTATCACAAGCGCGCGATAGAGAACTGGCACGACACCACTGTAGATTGTTCAGAAGATTCATTTTGGCTTGATGTCCAAGGTGACTCTATGACAGCACCGGCAGGGTTAAGCATTCCAGAAGGAATGATAATTCTGGTTGATCCCGAAGTCGAACCAAGAAACGGCAAGCTGGTTGTTGCAAAATTAGAAGGTGAAAACGAGGCCACATTCAAAAAATTAGTTATGGATGCAGGCCGAAAGTTTTTAAAACCATTAAACCCACAATATCCGATGATAGAAATCAACGGAAACTGCAAAATCATTGGCGTAGTTGTTGACGCAAAACTCGCAAATCTTCCATAG
TP901CI<br>5	AGTTCATGAAACGTGAACT	tactagaaagaggagaaatactagATGAAAACCGATACCAGTAACAGATTAAAACAAATAATGGCGGAACGCAACCTGAAACAGGTGGATATCTTAAACTTATCGATTCCATTTCAGAAAAAATTT

		GGTATCAAGCTGAGTAAAAGTACGCTGTCGCAGTATGTGAACTCTGTGCAGTCGCCAGACCAG<br>AACCGCATCTATTTACTGGCGAAAACGCTGGGCGTTTCCGAAGCTTGGCTGATGGGATTTGAT<br>GTTCCAATGGTTGAATCGTCGAAAATCGAAAACGATTCGGAAAACATCGAAGAAACCATTACG<br>GTGATGAAAAAATTGGAAGAGCCGAGACAGAAAGTGGTCCTGGATACCGCCAAAATTCAGCTG<br>AAAGAACAGGATGAGCAGAACAAAGTGAAACAGATCGAAGATTACCGTCTAAGTGATGAATAT<br>TTAGAAGAACAGATCAGTAAAGCGAGTGCATATGGAGGTGGCCAATTAAATGACAACGATAAA<br>GAATTTTTCAAACGTTTGTTGAAAAACACGTTAAAAGAAAAAATTGATAAAGGAGATCTGTGA
Cro<br>6	TATCACCGCAAGGGATA	tactagaaagcaggcaaatactagATGGAACAACGCATAACCCTGAAAGATTATGCAATGCGC<br>TTTGGGCAAACCAAGACAGCTAAAGATCTCGGCGTATATCAAAGCGCGATCAACAAGGCCATT<br>CATGCAGGCCGAAAGATTTTTTTAACTATAAACGCTGATGGAAGCGTTTATGCGGAAGAGGTA<br>AAGCCCTTCCCGAGTAACAAAAAAACAACAGCATGA
Mnt<br>7	AGGTCCACGGTGGACCT	tactagaaagatctataatactagATGGCCCGGGATGATCCTCACTTCAATTTTCGTATGCCA<br>ATGGAAGTAAGAGAGAAATTGAAATTTAGAGCAGAGGCAAACGGACGGAGCATGAACTCTGAG<br>CTTTTGCAAATCGTACAAGATGCCCTAAGCAAACCGTCACCAGTCACTGGGTACCGCAATGAT<br>GCGGAACGACTCGCCGATGAGCAGAGCGAGTTAGTGAAGAAGATGGTCTTCGATACACTGAAG<br>GATCTTTATAAAAAAACCACCTGA
TetR<br>8	TCCCTATCAGTGATAGA<br>GA	tactagaattagaaataatactagATGTCCAGATTAGATAAAAGTAAAGTGATTAACAGCGCA<br>TTAGAGCTGCTTAATGAGGTCGGAATCGAAGGTTTAACAACCCGTAAACTCGCCCAGAAGCTA<br>GGTGTAGAGCAGCCTACATTGTATTGGCATGTAAAAAATAAGCGGGCTTTGCTCGACGCCTTA<br>GCCATTGAGATGTTAGATAGGCACCATACTCACTTTTGCCCTTTAGAAGGGGAAAGCTGGCAA<br>GATTTTTTACGTAATAACGCTAAAAGTTTTAGATGTGCTTTACTAAGTCATCGCGATGGAGCA<br>AAAGTACATTTAGGTACACGGCCTACAGAAAAACAGTATGAAACTCTCGAAAATCAATTAGCC<br>TTTTTATGCCAACAAGGTTTTTCACTAGAGAATGCATTATATGCACTCAGCGCTGTGGGGCAT<br>TTTACTTTAGGTTGCGTATTGGAAGATCAAGAGCATCAAGTCGCTAAAGAAGAAAGGGAAACA<br>CCTACTACTGATAGTATGCCGCCATTATTACGACAAGCTATCGAATTATTTGATCACCAAGGT<br>GCAGAGCCAGCCTTCTTATTCGGCCTTGAATTGATCATATGCGGATTAGAAAAACAACTTAAA<br>TGTGAAAGTGGGTCCTGA
mTetR<br>9	TCCCCGTCAGTGACGGA<br>GA	tactagaaagaggagaaatactagATGATGAGCCGTCTCGACAAGTCAAAGGTTATAAATTCA<br>GCTCTCGAATTATTGAACGAAGTAGGTATAGAGGGGCTCACCACGAGAAAGTTGGCACAAAAA<br>TTGGGGGCTGCTCAAAAGACCCTCTACTGGCACGTTAAGAACAAAAGAGCACTCTTGGATGCA<br>CTCGCAATAGAAATGCTCGACCGTCATCACACGCATTTCTGTCCGCTCGAGGGTGAGTCATGG<br>CAGGACTTCCTCAGAAACAATGCAAAGTCATTCCGTTGCGCACTCTTGTCACACAGAGACGGT<br>GCTAAGGTTCACCTCGGGACCAGACCGACCGAGAAGCAATACGAGACGTTGGAGAACCAGCTC<br>GCATTCCTCTGTCAGCAGGGGTTCAGCTTGGAAAACGCTCTCTACGCTTTGTCAGCAGTTGGT<br>CACTTCACGCTCGGGTGTGTTCTCGAGGACCAGGAACACCAGGTAGCAAAGGAGGAGCGTGAG<br>ACCCCGACGACGGACTCAATGCCTCCTCTCCTCAGGCAGGCAATAGAGCTCTTCGACCATCAG<br>GGGGCTGAACCTGCATTTCTCTTTGGGTTGGAGCTCATAATCTGTGGTCTCGAGAAGCAGTTG<br>AAGTGCGAGTCAGGTAGCTGA
CymR<br>8	ACAAACAGACAATCTGG<br>TCTGTTTGTATT	tactagaaagctctctaatactagATGGTAATAATGAGCCCAAAAAGAAGAACACAAGCAGAA<br>AGAGCAATGGAAACACAAGGAAAACTAATAGCAGCAGCACTAGGAGTACTAAGAGAAAAAGGA<br>TACGCAGGATTCAGAATAGCAGACGTACCAGGAGCAGCAGGAGTAAGCAGAGGAGCACAAAGC<br>CACCACTTCCCAACAAAACTAGAACTACTACTAGCAACATTCGAATGGCTATACGAACAAATA<br>ACAGAAAGAAGCAGAGCAAGACTAGCAAAACTAAAACCAGAGGACGACGTAATACAACAAATG<br>CTAGACGACGCAGCAGAGTTCTTCCTAGACGACGACTTCAGCATAAGCCTAGACCTAATAGTA<br>GCAGCAGACCGCGACCCAGCACTAAGAGAAGGAATACAAAGAACAGTAGAAAGAAACAGATTC<br>GTAGTAGAGGACATGTGGCTAGGAGTGCTAGTAAGCAGAGGACTAAGCAGAGACGACGCAGAG<br>GACATACTATGGCTAATATTCAACAGCGTAAGAGGACTAGCAGTAAGAAGCCTATGGCAAAAA<br>GACAAAGAAAGATTCGAAAGAGTAAGAAACAGCACACTAGAAATAGCAAGAGAAAGATACGCA

		AAATTCAAAAGATGA
PhlF [8]	ATGATACGAAACGTACC<br>GTATCGTTAAGGT	tactagaaagaatagtaatactagATGGCACGTACCCCGAGCCGTAGCAGCATTGGTAGCCTG<br>CGTAGTCCGCATACCCATAAAGCAATTCTGACCAGCACCATTGAAATCCTGAAAGAATGTGGT<br>TATAGCGGTCTGAGCATTGAAAGCGTTGCACGTCGTGCCGGTGCAAGCAAACCGACCATTTAT<br>CGTTGGTGGACCAATAAAGCAGCACTGATTGCCGAAGTGTATGAAAATGAAAGCGAACAGGTG<br>CGTAAATTTCCGGATCTGGGTAGCTTTAAAGCCGATCTGGATTTTCTGCTGCGTAATCTGTGG<br>AAAGTTTGGCGTGAAACCATTTGTGGTGAAGCATTTCGTTGTGTTATTGCAGAAGCACAGCTG<br>GACCCTGCAACCCTGACCCAGCTGAAAGATCAGTTTATGGAACGTCGTCGTGAGATGCCGAAA<br>AAACTGGTTGAAAATGCCATTAGCAATGGTGAACTGCCGAAAGATACCAATCGTGAACTGCTG<br>CTGGATATGATTTTTGGTTTTTGTTGGTATCGCCTGCTGACCGAACAGCTGACCGTTGAACAG<br>GATATTGAAGAATTTACCTTCCTGCTGATTAATGGTGTTTGTCCGGGTACACAGCGTTGA
LmrA*	GATAATAGACCAGTCAC<br>TATATTT	tactagaaagaaagtcaatactagATGAGCTATGGTGATAGCCGTGAAAAAATTCTGAGCGCA<br>GCAACCCGTCTGTTTCAGCTGCAGGGTTATTATGGCACCGGTCTGAATCAGATTATCAAAGAA<br>AGCGGTGCACCGAAAGGTAGCCTGTATTATCATTTTCCGGGTGGTAAAGAACAGCTGGCAATT<br>GAAGCAGTGAACGAAATGAAAGAATATATCCGCCAGAAAATCGCCGATTGTATGGAAGCATGT<br>ACCGATCCGGCAGAAGGTATTCAGGCATTTCTGAAAGAACTGAGCTGTCAGTTTAGCTGTACC<br>GAAGATATTGAAGGTCTGCCGGTTGGTCTGCTGGCAGCAGAAACCAGCCTGAAAAGCGAACCG<br>CTGCGTGAAGCATGTCATGAAGCATATAAAGAATGGGCCAGCGTGTATGAAGAAAAACTGCGT<br>CAGACCGGTTGTAGCGAAAGCCGTGCAAAAGAAGCAAGCACCGTTGTTAATGCAATGATTGAA<br>GGTGGTATTCTGCTGAGCCTGACCGCAAAAAATAGCACACCGCTGCTGCATATTAGCAGCTGT<br>ATTCCGGATCTGCTGAAACGTGGTGGCGGTGGCAGCGGTGGCGGTGGCAGCAGATTTGTTGGG<br>CACGTTGAGCCCAAAGGGAAATATCCATTGATTAGCATGGTTAGAGCTGGTTCGTGGTGTGAA<br>GCTTGTGAACCCTACGATATCAAGGACATTGATGAATGGTATGACAGTGACGTTAACTTATTA<br>GGCAATGGATTCTGGCTGAAGGTTGAAGGTGATTCCATGACCTCACCTGTAGGTCAAAGCATC<br>CCTGAAGGTCATATGGTGTTAGTAGATACTGGACGGGAGCCAGTGAATGGAAGCCTTGTTGTA<br>GCCAAACTGACTGACGCGAACGAAGCAACATTCAAGAAACTGGTCATAGATGGCGGTCAGAAG<br>TACCTGAAAGGCCTGAATCCTTCATGGCCTATGACTCCTATCAACGGAAACTGCAAGATTATC<br>GGTGTTGTCGTGGAAGCGAGGGTAAAATTCGTATAA

[a] The underlined segments within promoter sequences indicate the promoter cores produced via genetic refinement.

[b] Protein coding sequences are indicated by capital letters. The CDS of an RNAP may be combined with different RBSs in different scenarios (See Supplementary Tables 5-7).

Supplementary Table 2 σ^{70}-dependent Promoters Emerging at the Interfaces of Transcriptional Elements.

Combined Elements	Promoter Prediction[a]
$O_{2/4\text{-AmtR}}$	TTTCTATCGATCTATAGATAATgctagctacTTTCTATCGATCTATAGATAATgctagctacTTTCTATCGATCTATAGATAATgctagctacTTTCTATCGATCTATAGATAATgctagctac
$O_{2/4\text{-LmrA*}}$	GATAATAGACCAGTCACTATATTTtagctacGATAATAGACCAGTCACTATATTTtagctacGATAATAGACCAGTCACTATATTTtagctacGATAATAGACCAGTCACTATATTTtagctac
P_{T7M43} + $O_{2\text{-cI434}}$	atgcctccacaccgctcgtcacatcctg**GACAACGACTCACTATAGGGG**TACAAGAAAGTTTGTTgctacTACAAGAAAGTTTGTTgctacTACAAGAAAGTTTGTTgctacTACAAGAAAGTTTGTTcgatg
P_{T7M48} + $O_{3\text{-TP901cI}}$	atgcctccacaccgctcgtcacatcctgAGTTCATGAAACGTGAACTatgctatg**CTCATCGACTCACTATAGGGG**AGTTCATGAAACGTGAACTtgccggtcgatc

[a] Operators are indicated by plain capital letters; promoter cores are indicated by bold capital letters. The -35 and -10 regions of putative σ^{70}-dependent promoters are underlined.

Supplementary Table 3. Parameter Database of Promoter Cores and Operators.

**Activator: T7 RNAP**		
**Promoter Cores**		α = 16,462; β=19; n_A = 1.34
**Name**	**Sequence**[a]	**[Activator]/K_A**
P_{T7wt}	TAATACGACTCACTATAGGGG	0.274 (K_A = 2,532)
P_{T7M1}	TATAACGACTCACTATAGGGG	0.115
P_{T7M2}	GAAAACGACTCACTATAGGGG	0.104
P_{T7M3}	GGATACGACTCACTATAGGGG	0.0382
P_{T7M4}	TAATACGACTCAGTCAAGGGG	0.0273
P_{T7M5}	GTGCACGACTCACTATAGGGG	0.00396
P_{T7M6}	TAATACGACTCACACTCGGGG	0.00185
P_{T7M11}	ATTTACGACTCACTATAGGGG	0.230
P_{T7M12}	AAATACGACTCACTATAGGGG	0.227
P_{T7M13}	GAATACGACTCACTATAGGGG	0.191
P_{T7M14}	TAAAACGACTCACTATAGGGG	0.160
P_{T7M15}	TTTAACGACTCACTATAGGGG	0.144
P_{T7M16}	CAAAACGACTCACTATAGGGG	0.142
P_{T7M17}	AATTCCGACTCACTATAGGGG	0.137
P_{T7M18}	AAAAACGACTCACTATAGGGG	0.124
P_{T7M19}	AGTTACGACTCACTATAGGGG	0.117
P_{T7M20}	CTAAACGACTCACTATAGGGG	0.0998
P_{T7M21}	ACTTACGACTCACTATAGGGG	0.0816
P_{T7M22}	TAAACCGACTCACTATAGGGG	0.0731
P_{T7M23}	ATAAACGACTCACTATAGGGG	0.0719
P_{T7M24}	GTAAACGACTCACTATAGGGG	0.0706
P_{T7M25}	AGATACGACTCACTATAGGGG	0.0643
P_{T7M26}	AATCACGACTCACTATAGGGG	0.0589
P_{T7M27}	TGATACGACTCACTATAGGGG	0.0565
P_{T7M28}	AGAAACGACTCACTATAGGGG	0.0518
P_{T7M29}	CTACACGACTCACTATAGGGG	0.0367
P_{T7M30}	CATCACGACTCACTATAGGGG	0.0332
P_{T7M31}	ATAAGCGACTCACTATAGGGG	0.0267
P_{T7M32}	ATACACGACTCACTATAGGGG	0.0256
P_{T7M33}	GCATTCGACTCACTATAGGGG	0.0247
P_{T7M34}	ATCTACGACTCACTATAGGGG	0.0244
P_{T7M35}	ACCTACGACTCACTATAGGGG	0.0212
P_{T7M36}	ACAAACGACTCACTATAGGGG	0.0204
P_{T7M37}	TCCTACGACTCACTATAGGGG	0.0203
P_{T7M38}	GCCTACGACTCACTATAGGGG	0.0199
P_{T7M39}	CACTACGACTCACTATAGGGG	0.0177
P_{T7M40}	GGCTACGACTCACTATAGGGG	0.0169
P_{T7M41}	TTGAACGACTCACTATAGGGG	0.0166
P_{T7M42}	CACAACGACTCACTATAGGGG	0.0161
P_{T7M43}	GACAACGACTCACTATAGGGG	0.0146
P_{T7M44}	TATCGCGACTCACTATAGGGG	0.0136
P_{T7M45}	CTCTGCGACTCACTATAGGGG	0.0113

P_{T7M46}	GTGAACGACTCACTATAGGGG	0.00962		
P_{T7M47}	CGCAACGACTCACTATAGGGG	0.00956		
P_{T7M48}	CTCATCGACTCACTATAGGGG	0.00804		
P_{T7M49}	TTTGTCGACTCACTATAGGGG	0.00614		
P_{T7M50}	AGCCACGACTCACTATAGGGG	0.00611		
P_{T7M51}	AGGAACGACTCACTATAGGGG	0.00575		
P_{T7M52}	TGCAGCGACTCACTATAGGGG	0.00372		
P_{T7M53}	ATAGACGACTCACTATAGGGG	0.00365		
P_{T7M54}	AAAGCCGACTCACTATAGGGG	0.00272		
P_{T7M55}	CCGTTCGACTCACTATAGGGG	0.00237		
P_{T7M56}	CGCACCGACTCACTATAGGGG	0.00235		
P_{T7M57}	GGTCGCGACTCACTATAGGGG	0.00231		
**Operators**				
**Name**	**Sequence**[b]	K_R	n_R	δ_R
$O_{1\text{-}cI434}$	[N$_{21}$]TACAAGAAAGTTTGTTcgatg	885	1.5	2811
$O_{2\text{-}cI434}$	[N$_{21}$]TACAAGAAAGTTTGTTgctacTACAAGAAAGTTTGTTgctacTACAAGAAAGTTTGTTgctacTACAAGAAAGTTTGTTcgatg	379	2	1141
$O_{3\text{-}cI434}$	TACAAGAAAGTTTGTTctatg[N$_{21}$]TACAAGAAAGTTTGTTcgatg	410	2.7	108
$O_{4\text{-}cI434}$	TACAAGAAAGTTTGTTctatg[N$_{21}$]TACAAGAAAGTTTGTTgctacTACAAGAAAGTTTGTTgctacTACAAGAAAGTTTGTTgctacTACAAGAAAGTTTGTTcgatg	350	3.8	64
$O_{1\text{-}HKcI}$	[N$_{21}$]TGAACCATAAGTTCAgctctg	771	0.9	193
$O_{2\text{-}HKcI}$	[N$_{21}$]TGAACCATAAGTTCAgctctgTGAACCATAAGTTCAgctctgTGAACCATAAGTTCAgctctgTGAACCATAAGTTCAgctctg	42	1.8	410
$O_{3\text{-}HKcI}$	TGAACCATAAGTTCAgctatg[N$_{21}$]TGAACCATAAGTTCAgctctg	91	2.7	25
$O_{4\text{-}HKcI}$	TGAACCATAAGTTCAgctatg[N$_{21}$]TGAACCATAAGTTCAgctctgTGAACCATAAGTTCAgctctgTGAACCATAAGTTCAgctctgTGAACCATAAGTTCAgctctg	50	3.2	0.1
$O_{1\text{-}P22c2}$	[N$_{21}$]ATTTAAGTGTTCTTTAATcgctgttccgctg	78	1.2	400
$O_{2\text{-}P22c2}$	[N$_{21}$]ATTTAAGTGTTCTTTAATcgctgttccgctgATTTAAGTGTTCTTTAATcgctgttccgctgATTTAAGTGTTCTTTAATcgctgttccgctgATTTAAGTGTTCTTTAATcgctgttccgctg	50	4.1	70
$O_{3\text{-}P22c2}$	ATTTAAGTGTTCTTTAATgagcatctgctatg[N$_{21}$]ATTTAAGTGTTCTTTAATcgctgttccgctg	52	2.6	191
$O_{4\text{-}P22c2}$	ATTTAAGTGTTCTTTAATgagcatctgctatg[N$_{21}$]ATTTAAGTGTTCTTTAATcgctgttccgctgATTTAAGTGTTCTTTAATcgctgttccgctgATTTAAGTGTTCTTTAATcgctgttccgctgATTTAAGTGTTCTTTAATcgctgttccgctg	42	4	47
$O_{1\text{-}TP901cI}$	[N$_{21}$]AGTTCATGAAACGTGAACTtgccggtcgatc	71	1.3	671
$O_{2\text{-}TP901cI}$	[N$_{21}$]AGTTCATGAAACGTGAACTtgccggtcgatcAGTTCATGAAACGTGAACTtgccggtcgatcAGTTCATGAAACGTGAACTtgccggtcgatcAGTTCATGAAACGTGAACTtgccggtcgatc	84	1.6	288
$O_{3\text{-}TP901cI}$	AGTTCATGAAACGTGAACTatgctatg[N$_{21}$]AGTTCATGAAACGTGAACTtgccggtcgatc	58	1.3	379
$O_{4\text{-}TP901cI}$	AGTTCATGAAACGTGAACTatgctatg[N$_{21}$]AGTTCATGAAACGTGAACTtgccggtcgatcAGTTCATGAAACGTGAACTtgccggtcgatcAGTTCA	78	1.5	207

	TGAAACGTGAACTtgccggtcgatcAGTTCATGAAACGTGAACTtgccggtcgatc			
$O_{1\text{-Cro}}$	[N_{21}]TATCACCGCAAGGGATAgatc	67	0.93	1897
$O_{2\text{-Cro}}$	[N_{21}]TATCACCGCAAGGGATAgatcTATCACCGCAAGGGATAgatcTATCACCGCAAGGGATAgatcTATCACCGCAAGGGATAgatc	82	1.1	1754
$O_{3\text{-Cro}}$	TATCACCGCAAGGGATActatg[N_{21}]TATCACCGCAAGGGATAgatc	70	0.71	1649
$O_{4\text{-Cro}}$	TATCACCGCAAGGGATActatg[N_{21}]TATCACCGCAAGGGATAgatcTATCACCGCAAGGGATAgatcTATCACCGCAAGGGATAgatcTATCACCGCAAGGGATAgatc	89	0.96	1600
$O_{1\text{-Mnt}}$	[N_{21}]AGGTCCACGGTGGACCTgatc	128	1.6	1136
$O_{3\text{-Mnt}}$	AGGTCCACGGTGGACCTctatg[N_{21}]AGGTCCACGGTGGACCTgatc	150	1.6	1218
$O_{1\text{-TetR}}$	[N_{21}]TCCCTATCAGTGATAGAGAtcacactccttc	18	2	81
$O_{4\text{-TetR}}$	tcTCCCTATCAGTGATAGAGAtcacactccttcaacctatg[N_{21}]TCCCTATCAGTGATAGAGAtcacactccttcTCCCTATCAGTGATAGAGAtcacactccttcTCCCTATCAGTGATAGAGAtcacactccttcTCCCTATCAGTGATAGAGAtcacactccttc	20	2.7	0.1
$O_{1\text{-mTetR}}$	[N_{21}]TCCCcgTCAGTGAcgGAGAtcacactccttc	69	0.7	0.1
$O_{3\text{-mTetR}}$	TCCCcgTCAGTGAcgGAGAtcacactctatg[N_{21}]TCCCcgTCAGTGAcgGAGAtcacactccttc	89	0.7	0.1
$O_{1\text{-CymR}}$	ACAAACAGACAATCTGGTCTGTTTGTATTactcaacctatg[N_{21}]ACAAACAGACAATCTGGTCTGTTTGTATTac	69	2	593
$O_{2\text{-CymR}}$	[N_{21}]ACAAACAGACAATCTGGTCTGTTTGTATTacACAAACAGACAATCTGGTCTGTTTGTATTacACAAACAGACAATCTGGTCTGTTTGTATTacACAAACAGACAATCTGGTCTGTTTGTATTac	88	2.3	270
$O_{3\text{-CymR}}$	ACAAACAGACAATCTGGTCTGTTTGTATTactcaacctatg[N_{21}]ACAAACAGACAATCTGGTCTGTTTGTATTac	89	2.3	511
$O_{4\text{-CymR}}$	ACAAACAGACAATCTGGTCTGTTTGTATTactcaacctatg[N_{21}]ACAAACAGACAATCTGGTCTGTTTGTATTacACAAACAGACAATCTGGTCTGTTTGTATTacACAAACAGACAATCTGGTCTGTTTGTATTacACAAACAGACAATCTGGTCTGTTTGTATTac	107	2.6	247
$O_{1\text{-PhlF}}$	[N_{21}]ATGATACGAAACGTACCGTATCGTTAAGGTc	630	2.8	123
$O_{2\text{-PhlF}}$	[N_{21}]ATGATACGAAACGTACCGTATCGTTAAGGTcATGATACGAAACGTACCGTATCGTTAAGGTcATGATACGAAACGTACCGTATCGTTAAGGTcATGATACGAAACGTACCGTATCGTTAAGGTc	731	2.9	0.1
$O_{3\text{-PhlF}}$	ATGATACGAAACGTACCGTATCGTTAAGGTcacaacctatg[N_{21}]ATGATACGAAACGTACCGTATCGTTAAGGTc	586	2.8	68
$O_{4\text{-PhlF}}$	ATGATACGAAACGTACCGTATCGTTAAGGTcacaacctatg[N_{21}]ATGATACGAAACGTACCGTATCGTTAAGGTcATGATACGAAACGTACCGTATCGTTAAGGTcATGATACGAAACGTACCGTATCGTTAAGGTcATGATACGAAACGTACCGTATCGTTAAGGTc	987	3.9	0.1
$O_{1\text{-LmrA*}}$	[N_{21}]GATAATAGACCAGTCACTATATTTtagctac	1670	2.4	485.7
$O_{3\text{-LmrA*}}$	GATAATAGACCAGTCACTATATTTtagctactcaacctatg[N_{21}]GATAATAGACCAGTCACTATATTTtagctac	1550	3.8	111.3

**Activator: σ^{ECF11}**	
**Promoter Cores**	α = 14,206; β =7.3; n_A = 1.13

Name	Sequence[a]	[Activator]/K_A		
$P_{ECF11wt}$	TGATCC[N16]CGTAACACCTCTG	0.376 (K_A = 73,425)		
$P_{ECF11M1}$	AGATCC[N16]CGTAACACCTCTG	0.178		
$P_{ECF11M2}$	CGATCC[N16]CGTAACACCTCTG	0.110		
$P_{ECF11M3}$	TGAGCC[N16]CGTAACACCTCTG	0.0557		
$P_{ECF11M4}$	TGCTCC[N16]CGTAACACCTCTG	0.0298		
$P_{ECF11M5}$	TGTTCC[N16]CGTAACACCTCTG	0.0108		
**Operators**				
**Name**	**Sequence**[c]	K_R	n_R	δ_R
$O_{1\text{-cI434}}$	actcttcatcccgcta[N13]TACAAGAAAGTTTGTTcgatg	720	1.5	232
$O_{2\text{-cI434}}$	actcttcatcccgcta[N13]TACAAGAAAGTTTGTTgctacTACAAGAAAGTTTGTTgctacTACAAGAAAGTTTGTTgctacTACAAGAAAGTTTGTTcgatg	263	2.0	94
$O_{3\text{-cI434}}$	TACAAGAAAGTTTGTT[N13]TACAAGAAAGTTTGTTcgatg	205	2.3	35
$O_{4\text{-cI434}}$	TACAAGAAAGTTTGTT[N13]TACAAGAAAGTTTGTTgctacTACAAGAAAGTTTGTTgctacTACAAGAAAGTTTGTTgctacTACAAGAAAGTTTGTTcgatg	155	2.4	12

[a] Mutations are shown in red.

[b] Single operators are indicated by capital letters. [N_{21}] indicates the random sequences to be replaced by the T7 promoter core.

[c] Sequence fragments upstream of [N_{13}] can be used to replace the [N_{16}] region in the ECF11 promoter core; sequence fragments downstream of [N_{13}] can be placed immediately downstream of the promoter core.

Supplementary Table 4. Sequences of Crucial Parts of the pPT and pRG Backbones.

Part Number[a]	Function	Sequence
I	Terminator	CTCGGTACCAAATTCCAGAAAAGAGGCCGCGAAAGCGGCCTTTTTTCGTTTTGG TCCTACTAGATGCCTCCACACCGCTCGTCACATCCTG
II	Golden Gate marker[b]	GgagaccTTACCATTCGCCATTCAGGCTGCGCAACTGTTGGGAAGGGCGATCGG TGCGGGCCTCTTCGCTATTACGCCAGCTGGCGAAAGGGGGATGTGCTGCAAGGC GATTAAGTTGGGTAACGCCAGGGTTTTCCCAGTCACGACGTTGTAAAACGACGG CCAGTGAATCCGTAATCATGGTCATAGCTGTTTCCTGTGTGAAATTGTTATCCG CTCACAATTCCACACAACATACGAGCCGGAAGCATAAAGTGTAAAGCCTGGGGT GCCTAATGAGTGAGCTAACTCACATTAATTGCGTTGCGCggtctcA
III	Ribozyme-based insulator	AGCTGTCACCGGATGTGCTTTCCGGTCTGATGAGTCCGTGAGGACGAAACAGCC TCTACAAATAATTTTGTTTAA
IV	Reporter gene[c]	tactagagaaagaggagaaatactagATGCGTAAAGGCGAAGAGCTGTTCACTG GTGTCGTCCCTATTCTGGTGGAACTGGATGGTGATGTCAACGGTCATAAGTTTT CCGTGCGTGGCGAGGGTGAAGGTGACGCAACTAATGGTAAACTGACGCTGAAGT TCATCTGTACTACTGGTAAACTGCCGGTACCTTGGCCGACTCTGGTAACGACGC TGACTTATGGTGTTCAGTGCTTTGCTCGTTATCCGGACCATATGAAGCAGCATG ACTTCTTCAAGTCCGCCATGCCGGAAGGCTATGTGCAGGAACGCACGATTTCCT TTAAGGATGACGGCACGTACAAAACGCGTGCGGAAGTGAAATTTGAAGGCGATA CCCTGGTAAACCGCATTGAGCTGAAAGGCATTGACTTTAAAGAAGACGGCAATA TCCTGGGCCATAAGCTGGAATACAATTTTAACAGCCACAATGTTTACATCACCG CCGATAAACAAAAAAATGGCATTAAAGCGAATTTTAAAATTCGCCACAACGTGG AGGATGGCAGCGTGCAGCTGGCTGATCACTACCAGCAAAACACTCCAATCGGTG ATGGTCCTGTTCTGCTGCCAGACAATCACTATCTGAGCACGCAAAGCGTTCTGT CTAAAGATCCGAACGAGAAACGCGATCATATGGTTCTGCTGGAGTTCGTAACCG CAGCGGGCATCACGCATGGTATGGATGAACTGTACAAATGA
V	Composite terminator	TGATAAGCCAGGCATCAAATAAAACGAAAGGCTCAGTCGAAAGACTGGGCCTTT CGTTTTATCTGTTGTTTGTCGGTGAACGCTCTCTACTAGAGTCACACTGGCTCA CCTTCGGGTGGGCCTTTCTGCGTTTATATACTAGAGCTGCTAACAAAGCCCGAA AGGAAGCTGAGTTGGCTGCTGCCACCGCTGAGCAATAACTAGCATAACCCCTTG GGGCCTCTAAACGGGTCTTGAGGGGTTTTTTGCTGAAAGGAGGAACTATATCCG GATTACTAGAGGTCATGCTTGCCATCTGTTTTCTTGCAAGAT
VI	Constitutively expressed *lac*I[c] (in reverse orientation)	TCACTGCCCGCTTTCCAGTCGGGAAACCTGTCGTGCCAGCTGCATTAATGAATC GGCCAACGCGCGGGGAGAGGCGGTTTGCGTATTGGGCGCCAGGGTGGTTTTTCT TTTCACCAGTGAGACTGGCAACAGCTGATTGCCCTTCACCGCCTGGCCCTGAGA GAGTTGCAGCAAGCGGTCCACGCTGGTTTGCCCCAGCAGGCGAAAATCCTGTTT GATGGTGGTTAACGGCGGGATATAACATGAGCTATCTTCGGTATCGTCGTATCC CACTACCGAGATATCCGCACCAACGCGCAGCCCGGACTCGGTAATGGCGCGCAT TGCGCCCAGCGCCATCTGATCGTTGGCAACCAGCATCGCAGTGGGAACGATGCC CTCATTCAGCATTTGCATGGTTTGTTGAAAACCGGACATGGCACTCCAGTCGCC TTCCCGTTCCGCTATCGGCTGAATTTGATTGCGAGTGAGATATTTATGCCAGCC AGCCAGACGCAGACGCGCCGAGACAGAACTTAATGGGCCCGCTAACAGCGCGAT TTGCTGGTGACCCAATGCGACCAGATGCTCCACGCCCAGTCGCGTACCGTCCTC ATGGGAGAAAATAATACTGTTGATGGGTGTCTGGTCAGAGACATCAAGAAATAA CGCCGGAACATTAGTGCAGGCAGCTTCCACAGCAATGGCATCCTGGTCATCCAG CGGATAGTTAATGATCAGCCCACTGACGCGTTGCGCGAGAAGATTGTGCACCGC CGCTTTACAGGCTTCGACGCCGCTTCGTTCTACCATCGACACCACCACGCTGGC

		ACCCAGTTGATCGGCGCGAGATTTAATCGCCGCGACAATTTGCGACGGCGCGTG<br>CAGGGCCAGACTGGAGGTGGCAACGCCAATCAGCAACGACTGTTTGCCCGCCAG<br>TTGTTGTGCCACGCGGTTGGGAATGTAATTCAGCTCCGCCATCGCCGCTTCCAC<br>TTTTTCCCGCGTTTTCGCAGAAACGTGGCTGGCCTGGTTCACCACGCGGGAAAC<br>GGTCTGATAAGAGACACCGGCATACTCTGCGACATCGTATAACGTTACTGGTTT<br>CATattcaccaccctgaattgactctcttccgggcgctatcatgccataccgcg<br>aaaggttttgcgccattcgatggcgcgccgc
VII	Inducible promoter[d]	TTCGTCAGGCCACATAGCTTTCTTGTTCTGATCGGAACGATCGTTGGCTGTGTT<br>GACAATTAATCATCGGCTCGTATAATGTGTGGAATTGTGAGCGCTCACAATT

[a] Neighboring parts were seamlessly ligated.

[b] *Bsa*I recognition sites are indicated with lower-case letters.

[c] The CDS is indicated with capital letters.

[d] The underlined regions show the -35, -10 regions and *lac*O sequence of P_{TAC}.

Supplementary Table 5. The Plasmids Used in Each Experiment in This Study

Experiment		Plasmid 1 (Cmr-pSC101)	Plasmid 2 (Ampr-P15A)	Cassette Integrated into Chromosome (via pOSIP-KO)	Inducer
Identification of insulated promoter cores	[a]T7 RNAP	pPT- library from saturation mutagenesis	pRGc	[b]P_{Sal}-RNAP	none
	[a]σ^{ECF11}			[c]P_c-RNAP	none
	σ^{ECF16}		pRG-RNAP	--	1 mM IPTG
	σ^{ECF20}				1 mM IPTG
	MmP1 RNAP				100 µM IPTG
	gh-1 RNAP				100 µM IPTG
	T3 RNAP				100 µM IPTG
Refinement of operators		pPT-operator	pRGc	--	none
Transcriptional activation	[IPTG]-Input curve	pPTc	pRGc	P_{TAC}-sfGFP	IPTG gradient
	[IPTG]-Output curve	pPT-promoter-core	pRGc	P_{TAC}-RNAP	IPTG gradient
Transcriptional repression	[IPTG]-Input curve	pPTc	pRG-sfGFP	$P_{C/TAC}$-RNAP	IPTG gradient
	[IPTG]-Output curve	pPT-promoter-operator	pRG-repressor	P_C-RNAP	IPTG gradient
IFFL		pPT-promoter-operator	pRG-repressor	P_{TAC}-RNAP	IPTG gradient

[a] These plasmid specifications were also used for the measurement of promoter core libraries.

[b] In this study, P_{Sal} was used as a constitutive promoter due to its high-level basal transcriptional activity.

[c] P_c: constitutive promoter.

Supplementary Table 6. Sequences of Cassettes integrated into the Chromosome of *E. coli* DH10B.

Cassette	Sequence[a]
P_{Sal}-T7 RNAP[b]	**gaattc**gcggccgcttctagagTCAATCCGTAAACAGGTCAAACATCAGTTGCCGCAACCAAATATTG<br>GCTAGGTCCTTGTGGTACTTCGCATGCCAGAACATGTTGATGGCTATTTCAGGCAAGACGACTGGGTG<br>CGGCAAGGCGCTTAGGCCGAAGGGCTCCACGCAGCAGTCGGCTAAACGTATCGGCACAGTGGCGAGCA<br>GATCGGTGCGCTGGAGGATGTGGCCAACGGCGGCGAAGTGCGGCACTTCCAGACGGATGTCGCGCCGG<br>ATGCCGACCCGTGTCATGTACGTGTCCACCTCGCCGTGGCCGGTGCCAGCGGCGATGACACGCACGTG<br>GCCGTAGGAACAGAAGCGCTCCAGAGTCAGGGGTTCGCGGGTGACTGGATGGTCCTTGCGACATAGGC<br>ACACGTAGTGATTCTGGAGCAGCCGGCGCTGAAAGAAGCCAGTTTGCAGATTGGGAAGCAGGCCCACG<br>GCCAAGTCCACGGTTCCGTTCTGCAAGGCCTGCATCAGGCTCATCGAACTGTCGCGCACCGTACTGAT<br>CACGCAATTGGGGGCCTGGTGAGCCAGCACATCCATCAGCCGCGGCATGAAGTAGATCTCGCCAATGT<br>CGGTCATGGCCAGGGTGAAGGTACGCTCGCTGGTCAGCGGATCGAAGCTTTCATGGTGCTGTAGGGCG<br>TTGCGCAGTGCGTGCATGGCCGAAGTGACGGGCTCGGCCAGATGCGCGGCATAGGGTGTGGGTTCCAT<br>TCCCTGATGTGTGCGCACGAAGAGTGGGTCCTGTAGCGAGGTGCGCAGGCGTTTCAGCGCATTGCTCA<br>CGGCAGGCTGGGTCAGGCCCAGGTTCTCCGCAGTGATAGAGACGCGTCTGTCGACCAGCAACTGGTTG<br>AACACCACCAGCAGGTTTAAATCCAGGTCACGCAGTTCCATggggcctcgcttgggttattgctggtg<br>cccggccgggcgcaatattcatgttgatgatttattatatatcgagtggtgtatttatcaatattgtt<br>tgctccgttatcgttattaacaagtcatcaataaagccatcacgag
P_{TAC}-sfGFP[c]<br>P_{TAC}-RNAP[c]	**gaattc**gcggccgcttctagagttcgtcaggccacatagctttcttgttctgatcggaacgatcgttg<br>gctgtgttgacaattaatcatcggctcgtataatgtgtggaattgtgagcgctcacaattagctgtca<br>ccggatgtgctttccggtctgatgagtccgtgaggacgaaacagcctctacaaataattttgtttaa
P_c-σ^{ECF11}	**gaattc**ttgacagctagctcagtcctaggtataatgctagcag
P_C-gh-1 RNAP[c]	**gaattc**ttgacggctagctcagtcctaggtacagtgctagcagctgtcaccggatgtgctttccggtc<br>tgatgagtccgtgaggacgaaacagcctctacaaataattttgtttaa
P_C-T3 RNAP[c]	**gaattc**ttgacggctagctcagtcctaggtatagtgctagcagctgtcaccggatgtgctttccggtc<br>tgatgagtccgtgaggacgaaacagcctctacaaataattttgtttaa

[a] The sequences of RNAP or ECF σ immediately downstream of these sequences are omitted for clarity. The *Eco*RI sites are shown in bold. The underlined sequences indicate the -35 and -10 regions of the promoters.

[b] The CDS of NahR is shown in capital letters.

[c] The sequence of RiboJ is included.

Supplementary Table 7. The RBS Sequences Used in Combination with RNAP Genes in Different Cassettes.

Cassette	RBS[a]
P_{TAC}-T7 RNAP	tactagagtatcagtaacagatactag
P_{TAC}-σ^{ECF11}	tactagagtcacacaggaaaggcctcg
pRG-gh-1 RNAP	Same as P_c-RNAP in Supplementary Table 1
pRG-T3 RNAP	Same as P_c-RNAP in Supplementary Table 1
pRG-MmP1 RNAP	As shown in Supplementary Table 1
pRG-σ^{ECF16}	
pRG-σ^{ECF20}	

[a] These sequences correspond to the sequences in shown in lower-case letters in Supplementary Table 1.

Supplementary References

1 Rhodius, V. A. *et al.* Design of orthogonal genetic switches based on a crosstalk map of sigmas, anti-sigmas, and promoters. *Molecular systems biology* **9**, 702, doi:10.1038/msb.2013.58 (2013).

2 Lou, C., Stanton, B., Chen, Y. J., Munsky, B. & Voigt, C. A. Ribozyme-based insulator parts buffer synthetic circuits from genetic context. *Nature biotechnology* **30**, 1137-1142, doi:10.1038/nbt.2401 (2012).

3 Carlson, N. G. *Characterization of the repressor from the lambdoid phage HK022* (The University of Arizona, Unpublished PhD dissertation, 1992).

4 Poteete, A. R. & Ptashne, M. Operator Sequences of Bacteriophages P22 and 21. *Journal of molecular biology* **137**, 81-91 (1980).

5 Pedersen, M., Ligowska, M. & Hammer, K. Characterization of the CI repressor protein encoded by the temperate lactococcal phage TP901-1. *J Bacteriol* **192**, 2102-2110, doi:10.1128/JB.01387-09 (2010).

6 A Johnson, B. J. M. & Ptashne, M. Mechanism of action of the cro protein of bacteriophage lambda. *PNAS* **75**, 1783-1787 (1978).

7 Vershon, A. K., Liao, S.-M., McClure, W. R. & Sauer, R. T. Bacteriophage P22 Mnt repressor DNA binding and effects on transcription in vitro. *Journal of molecular biology* **195**, 311-322 (1987).

8 Stanton, B. C. *et al.* Genomic mining of prokaryotic repressors for orthogonal logic gates. *Nat Chem Biol* **10**, 99-105, doi:10.1038/nchembio.1411 (2014).

9 Krueger, M., Scholz, O., Wisshak, S. & Hillen, W. Engineered Tet repressors with recognition specificity for the tetO-4C5G operator variant. *Gene* **404**, 93-100, doi:10.1016/j.gene.2007.09.002 (2007).
